# Validated Screening Tools for Common Mental Disorders in Low and Middle Income Countries: A Systematic Review

**DOI:** 10.1371/journal.pone.0156939

**Published:** 2016-06-16

**Authors:** Gemma-Claire Ali, Grace Ryan, Mary J. De Silva

**Affiliations:** 1 Department of Public Health and Primary Care, University of Cambridge, Cambridge, United Kingdom; 2 Centre for Global Mental Health, London, United Kingdom; 3 Department of Population Health, London School of Hygiene & Tropical Medicine, London, United Kingdom; 4 Department of Population, Environment and Health, Wellcome Trust, London, United Kingdom; University of Kwazulu-Natal, SOUTH AFRICA

## Abstract

**Background:**

A wide range of screening tools are available to detect common mental disorders (CMDs), but few have been specifically developed for populations in low and middle income countries (LMIC). Cross-cultural application of a screening tool requires that its validity be assessed against a gold standard diagnostic interview. Validation studies of brief CMD screening tools have been conducted in several LMIC, but until now there has been no review of screening tools for all CMDs across all LMIC populations.

**Methods:**

A systematic review with broad inclusion criteria was conducted, producing a comprehensive summary of brief CMD screening tools validated for use in LMIC populations. For each validation, the diagnostic odds ratio (DOR) was calculated as an easily comparable measure of screening tool validity. Average DOR results weighted by sample size were calculated for each screening tool, enabling us to make broad recommendations about best performing screening tools.

**Results:**

153 studies fulfilled our inclusion criteria. Because many studies validated two or more screening tools, this corresponded to 273 separate validations against gold standard diagnostic criteria. We found that the validity of every screening tool tested in multiple settings and populations varied between studies, highlighting the importance of local validation. Many of the best performing tools were purposely developed for a specific population; however, as these tools have only been validated in one study, it is not possible to draw broader conclusions about their applicability in other contexts.

**Conclusions:**

Of the tools that have been validated in multiple settings, the authors broadly recommend using the SRQ-20 to screen for general CMDs, the GHQ-12 for CMDs in populations with physical illness, the HADS-D for depressive disorders, the PHQ-9 for depressive disorders in populations with good literacy levels, the EPDS for perinatal depressive disorders, and the HADS-A for anxiety disorders. We recommend that, wherever possible, a chosen screening tool should be validated against a gold standard diagnostic assessment in the specific context in which it will be employed.

## Introduction

### The Importance of Common Mental Disorders

The World Health Organisation (WHO) International Classification of Disease (ICD-10) defines common mental disorders (CMDs) as ‘mood disorders’ and ‘neurotic, stress-related and somatoform disorders’ [1]. These include depressive, anxiety and post-traumatic stress disorders (PTSD). CMDs have been shown to negatively impact a wide range of health, economic and social outcomes [2, 3]. Co-morbidity with other health problems is high and worsens prognosis [4–6], and prevalence of CMDs is higher in low-income groups [7], ethnic minorities [8] and migrant populations [9, 10]. Both researchers and clinicians are therefore increasingly interested in screening for anxiety, depression and PTSD in the populations they study and treat.

### The Importance of Screening Tools

Brief screening tools are essential for improving mental health care in low and middle income countries (LMIC). The majority of health workers have neither the time nor training to administer complex diagnostic interviews to all individuals at risk of psychiatric illness. Adopting appropriate screening instruments is therefore an important first step to integrate care for CMDs into existing primary health care (PHC) services, particularly those attended by high risk populations, such as HIV or maternity clinics [11].

Brief CMD screening tools can also be used to enhance research and training in LMIC. The availability of short, simple tools will encourage researchers to screen for CMDs in their study populations, facilitating research into the effects of untreated mental illness on priority health, economic and social issues. Screening tools can also be used as part of a mental health training package for PHC workers [12]. By providing a succinct overview of symptoms, they teach health workers what to look for and thus improve their ability to detect mental health problems.

### The Importance of Cultural Validation

A wide range of screening tools is available to detect CMDs, but few have been specifically developed for LMIC populations. There is concern that using tools developed for high-income country populations will miss cases in LMIC. In sub-Saharan Africa, for example, distress is believed to be more commonly expressed through somatic symptoms and local idioms [13]. Although CMDs are prevalent in all regions worldwide, clinical presentation does differ between settings [14]. For example, previous validations of the Edinburgh postnatal depression scale (EPDS) in LMIC have generally found lower optimum cut-off scores than those recommended for the populations in which the tools were developed [15]. This may be due to cross-cultural differences in somatization of symptoms and expression of emotional distress, leading to under-recognition or misidentification of psychiatric morbidity [13].

Screening tool validity is the extent to which an instrument measures what it claims to measure. For cross-cultural application of a screening tool, it is most important to assess criterion validity [16]. This involves comparing the results of a screening tool to those of a recognised gold standard, defined as ‘a relatively irrefutable standard that constitutes recognized and accepted evidence that a certain disease exists’ [17]. The most reliable gold standards employed in cross-cultural mental health research are diagnostic interviews conducted by qualified mental health professionals [16].

### The Importance of this Systematic Review

Validation studies of brief CMD screening tools have been conducted in several LMIC, but significant gaps remain. Until now there has been no pooled resource from which researchers or implementers can identify the best-performing tools for their needs. Several smaller reviews have been conducted of screening tool validation studies for particular disorders in particular settings or populations–such as perinatal depression in Africa [18], depression in Spanish-speaking populations [19], and depression in Chinese older adults [20]–but to date there has been no review of screening tools for all CMDs across all LMIC populations. The project presented here updates and builds upon a 2012 systematic review of depression (as opposed to all CMD) screening tools validated in LMIC [21], which identified 19 studies.

By conducting a comprehensive systematic review of studies validating brief CMD screening tools for use in LMIC, we provide researchers, policy makers and health care providers with a comprehensive, evidence-based summary of the most appropriate CMD screening tools for use in particular settings and populations. We aim to provide a ‘one-stop shop’ at which researchers can compare the performance of screening tools in settings and populations similar to those in which they work, and identify appropriate cut-off points for probable diagnosis of the disorders of interest. A copy of the results database is available as a web appendix (S1 File), on figshare and on the Mental Health Innovation Network (MHIN), presenting the results of all validation studies identified by this review.

### Study Aim

We aimed to conduct a high quality systematic review of studies validating brief CMD screening tools for use in LMIC populations, using criterion validity as the outcome measure and gold standard diagnostic assessment as the comparison.

## Methods

We did not publish a study protocol in advance of conducting this review, but no methodological changes were made between study conception and completion.

### Search Strategy

**Fig 1:**
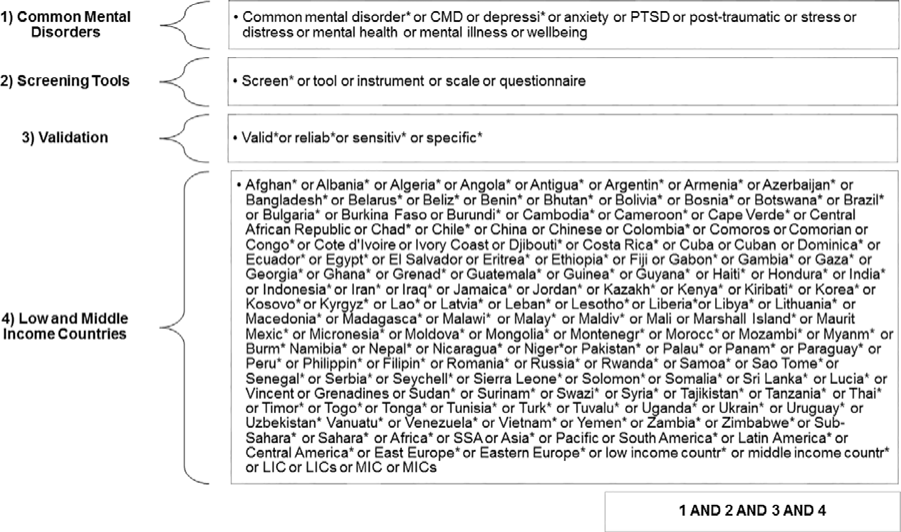
Database search strategy.

The search terms listed in Fig 1 were used to identify relevant papers from the EMBASE, Global Health, MEDLINE, PsychExtra and PsychInfo databases. The search was run on 11^th^ December 2013 and results were not restricted by publication date or language.

### Study Selection

All abstracts returned by the database search were reviewed for possible inclusion. Full texts were retrieved for those identified as potentially relevant, and these were assessed for inclusion using the criteria below. The reference lists of all studies that met these criteria were then used to identify additional studies for inclusion, as were all systematic reviews identified by the initial search. To reduce bias caused by human error, the second author repeated 10% of the study selection process at every stage. Rates of agreement were consistently high between the two reviewers, with any discrepancies resolved through discussion.

### Inclusion Criteria

#### Study design

Papers were eligible for inclusion in this review if they reported a criterion validation study of one or more screening tools.

#### Disorders

We included screening tool validation studies for any CMD included in the World Health Organisation’s International Classification of Diseases, version 10 [1].

#### Low and Middle Income Countries

Studies were included if conducted in a LMIC, as defined by the World Bank’s country classification [22].

#### Gold standard

To be eligible for inclusion, the study must have compared the screening tool’s performance with that of a recognised gold standard. The preferred gold standard was diagnostic assessment by a mental health professional. Where the gold standard diagnosis was made by a lay interviewer or general medical professional, the study was deemed acceptable only if a well-structured diagnostic interview suitable for delivery by a non-mental health professional was employed.

### Exclusion Criteria

In order to maximise the breadth of the review, all studies which met the above inclusion criteria were included irrespective of their methodological quality (see below). All study settings and population groups were included and details of each study are fully reported in the data extraction tables. This approach was designed to maximise the utility of the study findings. It enables researchers and health care providers to consider the full range of circumstances for which tools have been validated, and to identify which tools perform best in the settings and populations that best reflect their own research or clinical context.

### Quality Appraisal

Study quality was assessed against a modified version of Greenhalgh’s ten-item checklist for papers reporting validations of diagnostic or screening tests [23]. The final quality criteria employed for this review are presented in Table 1 below.

**Table 1:** Quality Criteria.

1	Was expectation bias avoided?
	(Were people administering the diagnostic interview blind to the results of the screening tool, and vice versa?)
2	Was work-up bias avoided?
	(Did positive and negative screens have an equal chance of receiving the full diagnostic interview?)
3	Was a sensible ‘normal range’ derived from the results?
	(Was ROC analysis used to identify the most appropriate cut-off point?)
4	Was the tool appropriately translated, adapted and/or designed for the study setting and population?
	(If using an existing tool, did authors employ the standardized WHO translation protocol? [24])
5	Were confidence intervals given for AUC, sensitivity, specificity and other psychometric features of the test?
6	Was the tool shown to be reproducible both within and/or between observers?
	(Was test-retest and/or inter-rater reliability assessed?)

Studies that met all the quality criteria were considered to be of ‘very good’ quality. Those that met criteria 1, 2 and 3 and at least one of criteria 4, 5 and 6 were classed as ‘good’ quality. Studies were classified as ‘fair’ quality if they failed to avoid work-up bias, or if they avoided work-up bias but did not meet any of criteria 4 to 6. Those that did not perform receiver operator characteristic curve (ROC) analysis to identify the most appropriate cut-off point were classified as ‘acceptable’ quality. Studies in which administrators of the screening tool and diagnostic interview were not blinded to each other’s results, or for which we were unable to ascertain whether this was the case, were recorded as unblinded.

### Data Extraction

Data extraction forms were piloted and finalised in March 2014. Data were extracted on disorders, screening tools, gold standards, tool administrators, study settings, study populations, sample sizes and psychometric properties of the screening tools. The key measures of screening tool performance were area under the receiver operating characteristic curve (AUC), sensitivity and specificity. Where available, data were also extracted on predictive values, correct classification rate, internal consistency (Cronbach’s alpha coefficient) and test-retest reliability. Where data were not reported in the published paper, authors were contacted by email. Information obtained in this manner was added to the data table available as a web appendix (S1 File), on figshare and on MHIN.

### Data Analysis

For each validation, the diagnostic odds ratio (DOR) [25] was calculated using the following formula:

$$DOR=\frac{Sensitivity/(100-Sensitivity)}{(100-Specificity)/Specificity}$$

DOR is a measure of screening tool effectiveness. It is defined as the ratio of the odds of a true positive screening positive relative to the odds of a true negative screening positive. Possible results range from 0 to infinity, with higher ratios indicating a better performing test. DOR increases very steeply as sensitivity and specificity tend towards 100%, so the following cut-offs were applied to rate screening tool validity: DOR≥50 for very strong validity, 50>DOR≥20 for strong, 20>DOR≥10 for fair and 10>DOR for weak. Although the same DOR can correspond to different combinations of sensitivity and specificity, it provides an acceptable comparison of the tools included in this review, because for each validation we have reported psychometric properties of the tool at the cut-off point that best balances sensitivity and specificity, as determined by the ROC analysis [25].

Average DOR results weighted by sample size were calculated for each screening tool to compare their effectiveness. Where a study reported significantly higher DOR than the norm, a second weighted average was calculated to exclude the outlier and produce a more reliable estimate of the screening tool’s validity in most LMIC settings and populations.

## Results

### Study Selection

**Fig 2:**
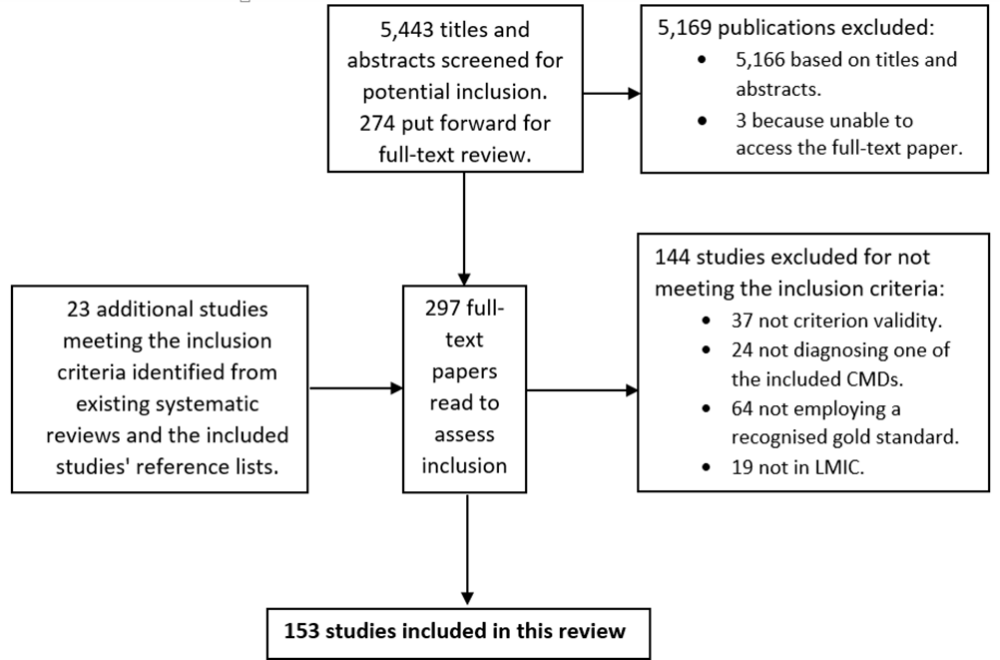
Study selection flow diagram.

The initial database search returned 5,443 original hits, from which 277 studies were identified as potentially relevant from their titles and abstracts. 274 full-text papers were retrieved and 130 of these met the criteria for inclusion. The reference lists of these 130 papers were then scanned for additional relevant studies not identified by the initial search. This, along with assessment of four existing systematic reviews [18–21], provided an additional 23 studies for inclusion. The total number of included studies was therefore 153. Fig 2 provides a flow diagram of this process, and a full list of included studies with references is available as a web appendix (S2 File) and on MHIN.

### Quality Appraisal

12 studies met all six quality criteria and were therefore classified as ‘very good’ quality. 88 studies were of ‘good’ quality, with the most commonly missed criteria being failure to report confidence intervals (criteria 5) and no attempt to assess the tool’s reproducibility (criteria 6). 25 studies were classed as ‘fair’ quality; 12 because they failed to avoid work-up bias and a further 13 because they met this criteria but did not meet criteria 4, 5 or 6.

11 studies were considered to be of ‘acceptable’ quality due to failure to conduct a ROC analysis to identify the tool’s optimum cut-off point for the population of interest. 17 studies were recorded as ‘unblinded’ (4 confirmed that the tool administrators were not blinded; 13 did not report on blinding and did not respond when contacted). A detailed breakdown of this quality appraisal is available as an online appendix (S3 File) and on MHIN.

### Description of Included Studies

Because several studies validate multiple tools, the 153 included studies correspond to 273 screening tool validations. Of these, 61 validate tools for any CMD, 175 for depressive disorders, 24 for anxiety disorders, and 13 for PTSD. Table 2 presents the CMDs for which screening tool validation studies were identified for this review.

**Table 2:** Disorders for which screening tools were validated by studies included in this review.

**Common Mental Disorders**	Any common mental disorder
(61 validations)	
	Dysthemia
	Any depressive disorder
**Depressive Disorders**	Major depressive disorder
(175 validations)	Antenatal depression
	Postnatal depression
	Postnatal major depressive disorder
	Any anxiety disorder
**Anxiety Disorders**	Generalised anxiety disorder
(24 validations)	Social anxiety disorder
	Panic disorder
**Post-traumatic stress disorder**	Post-traumatic stress disorder
(13 validations)	

Table 3 describes the populations sampled in the included studies. Perinatal women and people attending primary care clinics were particularly well-represented groups, while children and adolescents were under-represented. Of the 153 included studies, only 17 (11%) validated screenings tools to identify CMDs in children or adolescents, though in LMIC the proportion of the total population under age 15 ranges from 20 to 50%.

**Table 3:** Number of validation studies for each population group.

Population		Number of Studies
Child & Adolescent		17
Perinatal	Antenatal	9
	Postnatal	27
	Clinic attendees	34
	HIV+	6
	Physically ill	9
Adult	Mentally ill	8
	Trauma survivors	5
	General population	20
	University students	8
Elderly		15
		158
Total		(5 of the 153 studies sampled from populations
		representing two of the above groups)

Of the 153 studies eligible for inclusion in this review: 66 were conducted in Asia, 46 in South and Central America, 40 in Africa, and just 3 in East and Central Europe. This implies a geographically uneven distribution of research into CMD screening tool validity in LMIC, which becomes far more pronounced when looking within continents. For example, of the 43 South American studies meeting the inclusion criteria, over three quarters (33) were conducted in Brazil. Table 4 highlights this and other disparities in the geographic distribution of existing evidence, including the number of countries in each region for which no study was identified.

**Table 4:** Number of validation studies by subregion and country.

Region (# studies)		Countries Included (# studies)
	Central (1)	Cameroon (1)
		7 LMIC countries with 0 studies
	East (19)	Burundi (2), Ethiopia (3), Malawi (1), Rwanda (1), Somalia (1), Uganda (4), Zambia (4), Zimbabwe (3)
		11 LMIC countries with 0 studies
Africa	North (3)	Egypt (2), Morocco (1)
(40)		5 LMIC countries with 0 studies
	South (8)	Botswana (1), South Africa (7)
		3 LMIC countries with 0 studies
	West (9)	Burkina Faso (1), Nigeria (8)
		15 LMIC countries with 0 studies
	Caribbean (0)	17 LMIC countries with 0 studies
America	Central (3)	Honduras (1), Mexico (2)
(46)		6 LMIC countries with 0 studies
	South (43)	Brazil (33), Chile (1), Colombia (8), Peru (1)
		7 LMIC countries with 0 studies
	Central (1)	Tajikistan (1)
		4 LMIC country with 0 studies
	East (16)	China (15), Mongolia (1)
		1 LMIC country with 0 studies
Asia	South (23)	Bangladesh (1), India (12), Iran(4), Nepal (2), Pakistan (1), Sri Lanka (3)
(66)		3 LMIC countries with 0 studies
	Southeast (18)	Malaysia (7), Thailand (7), Vietnam (4)
		6 LMIC countries with 0 studies
	West (8)	Lebanon (3), Turkey (5)
		8 LMIC countries with 0 studies
Europe	East (0)	6 LMIC countries with 0 studies
(1)	South(1)	Bosnia & Herzegovina (1)
		5 LMIC countries with 0 studies
Oceania (0)		16 LMIC countries with 0 studies
**Total (153)**		**102 LMIC countries with 0 studies**

### Summary of Screening Tool Validity

Although it would be inappropriate to recommend ‘best’ screening tools for use in LMIC, we can make broad statements about the tools’ psychometric properties and their overall relative performance. Table 5 presents the weighted diagnostic odds ratio (DOR) of screening tools for which more than one study examined the tool’s ability to screen for a particular diagnosis. Table 5 categorises the validity of these screening tools according to their diagnostic odds ratio: DOR≥50 for very strong validity, 50>DOR≥20 for strong, 20>DOR≥10 for fair and 10>DOR for weak.

**Table 5:** Weighted diagnostics odds ratios (DORs) of selected screening tools.

Screening Tool	Disorder	Number of Studies	Weighted DOR (excluding outliers)
*GHQ-5*	*CMD*	*2*	*59.82*
**SRQ-20**	**CMD**	**14**	**28.36**
**GHQ-12**	**CMD**	**13**	**22.59**
K-6	CMD	2	15.61
K-10	CMD	2	15.55
GHQ-28	CMD	2	15.31
*SSQ*	*CMD*	*2*	*9*.*37*
*EPDS*	*CMD*	*3*	*7*.*01*
**HADS-D**	**Depressive disorders**	**8**	**33.07**
**PHQ-9**	**Depressive disorders**	**5**	**27.52**
**PHQ-2**	**Depressive disorders**	**2**	**22.18**
BDI	Depressive disorders	2	16.14
*EPDS*	*Depressive disorders*	*3*	*4*.*95*
**ZSDS**	**Major depressive disorder**	**4**	**36.47**
**GDS-15**	**Major depressive disorder**	**4**	**31.97**
**HADS-D**	**Major depressive disorder**	**3**	**22.77**
PHQ-9	Major depressive disorder	11	19.22
CES-D	Major depressive disorder	6	18.79
BDI	Major depressive disorder	4	15.18
*K-10*	*Major depressive disorder*	*3*	*8*.*58*
*EPDS*	*Postnatal major depressive disorder*	*4*	*172.70*
*EPDS*	*Postnatal depression*	*13*	*148.68*
EPDS	Antenatal depression	4	16.14
*HADS-A*	*Anxiety disorders*	*8*	*60.09*

Screening tool validity = *Very strong*, **strong**, fair, *weak*

Although not shown in Table 5, as they were only validated in one study each, many of the best performing tools included in this review are those developed from scratch for specific populations in particular settings. This process of tool development consists of either interviews with patients or analysis of psychiatric case notes to identify common idioms and symptom expression of CMDs in the local context. The following tools all performed with DORs of well over 100: case description for CMDs in China [26], the Chinese Military Mental Health Scale (CMMHS) for CMDs [27], and the Pakistan Anxiety and Depression Questionnaire (PADQ) [28]. The first two of these results come from good quality studies, though the third is of acceptable quality. A full table of results, including diagnostic odds ratios for all screening tool validations, is available as a web appendix (S1 File), on figshare and on MHIN.

## Discussion

### Choosing a Screening Tool

Although this review provides a comprehensive summary of the existing literature, and can therefore recommend screening tools that are likely to perform well in a given setting, local validation should still be conducted wherever possible. Where the resources exist to do so, a pilot study should always be carried out to validate the chosen screening tool against a gold standard diagnostic interview, confirming its validity for the study population.

Many of the best performing tools included in this review are those that were locally adapted. Where possible, a screening tool’s validity should therefore always be improved through local adaptation. Focus group discussions with representatives of the population in which the screening tool is to be implemented should be conducted, with two key aims. The first is to ensure that all questions are correctly understood and that none cause any discomfort to either interviewers or respondents. The second is to better understand local experience and expression of mental illness, allowing for local idioms of distress to be incorporated into the questionnaire.

#### Screening Tools for any CMD

We identified validation studies for 25 different tools screening for any CMD (including variants on the same baseline tool in terms of language or number of items). Of these, the **GHQ-5/12** and **SRQ-20** demonstrate the strongest psychometric properties. The **SRQ-20**’s binomial response format makes it particularly valuable for CMD screening in LMIC, as it can be effectively administered by lay interviewers with only minimal training, as well as easily understood and completed by respondents with low literacy [29]. The **GHQ-12** and **HADS** are particularly appropriate for detecting psychiatric morbidity in physically ill patients because, unlike the GHQ-30, they do not include questions about somatic symptoms [30].

#### Screening Tools for Depressive Disorders

Validation studies were found for 63 different tools screening for depressive disorders. The **PHQ-9** is one of the most commonly used depression screening tools, perhaps because it was the first to efficiently establish psychiatric diagnoses based on DSM-IV criteria. This review’s findings, however, do not strongly support its use in low resource LMIC settings. The **PHQ-9** performs very well in two large studies conducted in university student populations [31, 32], but poorly in several clinic populations with lower average education [33–35]. We therefore recommend the **PHQ-9** to screen for depressive disorders in high literacy population groups, but suggest that it may not be an appropriate tool for people with low literacy. After excluding outlying results, we found that the average performance of the HADS-D was slightly better than that of the PHQ-9, therefore also recommend the **HADS-D** for consideration when selecting a screening tool for depression.

The **EPDS** consistently performs very well as a screen for postnatal depression. Two qualities that make it particularly suitable for use in LMIC are its brevity and avoidance of the word ‘depression’ [36]. Comprising just ten items, the **EPDS** is relatively quick to complete and can be easily incorporated into existing postnatal services. We therefore strongly recommend the **EPDS** to screen for depression in postnatal women, but it performs much less well in other populations.

#### Screening Tools for Anxiety Disorders

This review finds that 11 different anxiety disorder screening tools have been validated for use in LMIC. Of these, the **HADS-A** performs notably better than the others. The **HADS** is unusual in its ability to detect specific mood states, and we particularly recommend its anxiety subscale (**HADS-A**) as a screen for anxiety disorders.

#### Screening Tools for PTSD

The evidence on screening tool validity for PTSD in LMIC is very scarce. This is particularly concerning given the research and clinical interest in the traumatic effects of humanitarian crises. Studying these issues requires measurement methods that have been appropriately validated for these intensely vulnerable populations. We identified validity studies for ten PTSD screening tools in LMIC. In the absence of sufficient evidence, we only provisionally recommend continued use of what is currently the most widely validated tool in LMIC, the HTQ (separate validation studies have been conducted in South Africa, Bosnia and Herzegovina, and Tajikistan).

### Strengths and Limitations of this Review

This study employed robust methods, such as 10% double screening at every stage of the search, and the use of standardised quality assessment criteria. Five databases were systematically searched and all potentially relevant papers were read in full. The decision to work with relatively limited exclusion criteria has maximised the scope of the evidence covered here for reference by researchers, policy makers and health workers.

On the other hand, inclusion was restricted to studies that validated the screening tool against a gold standard diagnostic interview. As with the brief screening tools we are interested in validating, these gold standards were developed for use in high income country populations. It is important to consider whether it is appropriate to treat them as a true gold standard for diagnosing CMDs in LMIC, and the extent to which this might limit our findings.

The validity of the conclusions drawn from this review is limited by the quality of the included studies. We were not able to calculate confidence intervals for weighted diagnostic odds ratios as very few of the included studies reported confidence intervals for sensitivity and specificity. That some studies failed to guard against expectation or work-up bias is also cause for concern. Although sample size was integrated into the average DOR calculations, overall quality scores were not taken into account, which we recognise as a limitation. We were also unable to conduct sensitivity analyses to explore the effect of study quality, due to the low number of studies for each tool and the large amount of between-study heterogeneity. Instead, full details of each study’s quality are provided as an online appendix (S3 File) and on MHIN, allowing readers to consider the quality of the individual studies conducted in the contexts that best reflect their own. As well as the risk of bias within studies, there is a risk of publication bias across studies due to the tendency to over-report positive findings, though this bias is likely to be the same for all screening tools and therefore should have little effect on relative validity.

Several additional studies were identified through a hand search, suggesting a limitation in the search strategy. Although this was developed in consultation with a qualified librarian, it may be necessary for future reviews to adapt the search terms in order to improve sensitivity. Possible issues to keep in mind include the varied terminology used to describe common mental disorders.

### Recommendations for Future Research

It is difficult to draw reliable conclusions from such a heterogeneous set of studies. In order to facilitate future reviews comparing screening tool validity, a set of methodological standards should be agreed upon for validation studies. We suggest that the screening tool should be conducted by a lay interviewer or general health worker, and the gold standard diagnostic interview by a mental health professional. In order to address concerns about the validity of so-called ‘gold standards’, we also recommend the validation of diagnostic interviews in LMIC populations.

This review employed broad inclusion criteria and relaxed quality criteria, thus identifying 153 studies for inclusion. Despite the large total number of studies, there are significant biases in the existing evidence. While there is plentiful evidence for screening tool validity for particular disorders in particular settings–for example depression in Brazil–huge gaps remain. There are over 100 LMIC for which no CMD screening tool validation study was identified for inclusion in this review. Further research is required to test the validity of screening tools in most LMIC settings and populations, particularly in those countries for which no validation studies have been conducted. There is a particular shortage of studies validating tools for use in community populations [31], and this review highlights a lack of attention to child and adolescent mental health. Screening tools for depression have been much more widely validated than those for anxiety and PTSD, or even for common mental disorders more broadly. These gaps should be made priorities for future research. We also recommend a shift in focus away from screening tools for narrowly defined disorders, and encourage the development of better transdiagnostic screens.

## Conclusions

Our results reinforce the importance of validating brief CMD screening tools for the particular populations and settings in which they are being applied. They demonstrate that a screening tool’s ability to accurately detect CMDs can vary significantly depending on the population in which it is administered, as can the most appropriate cut-off point for positive/negative classification. Our primary recommendation is that, wherever possible, a chosen screening tool should be validated against a gold standard diagnostic assessment in the specific context. Where this is not possible, health care professionals, researchers and policymakers can refer to the database of validation studies provided in the web appendix (S1 File), on figshare and on MHIN, to identify previous studies conducted in the region, country, population group and research setting of interest.

Many of the best performing tools were developed or adapted for specific populations; however, as these tools were only validated in one study, it is not possible to draw broader conclusions about their applicability in other contexts. Of the tools that have been validated in multiple settings, the authors broadly recommend using the SRQ-20 to screen for general CMDs, the GHQ-12 for CMDs in people with physical illness, the HADS-D for depressive disorders, the PHQ-9 for depressive disorders in populations with good literacy levels, the EPDS for perinatal depressive disorders, and the HADS-A for anxiety disorders.

## Supporting Information

S1 File Results Database. Complete database of results extracted from the included studies.
(XLSX)

S2 File Included Studies. Basic characteristics and references for all included studies.
(DOCX)

S3 File Quality Appraisal. Quality criteria met and missed by each of the included studies.
(XLSX)

S4 File Screening Tool Acronyms. Acronyms for all screening tools listed in the results database.
(DOCX)

S5 File PRISMA Checklist. (DOCX)
