# Supplementary material for: Validated Screening Tools for Common Mental Disorders in Low and Middle Income Countries: A Systematic Review
Source: PLoS One. 2016 Jun 16;11(6):e0156939. doi: 10.1371/journal.pone.0156939 (PMC4911088; doi:10.1371/journal.pone.0156939)
Supplement: S2 File — Basic characteristics and references for all included studies. (DOCX) [file pone.0156939.s002.docx]

**S2 File. Included Studies**

| **Author** | **Disorders** | **Screening Tools** | **Country** | **Populations** |
| --- | --- | --- | --- | --- |
| Abeyasinghe et al., 2012) [1] | Major depressive episode | PDS | Sri Lanka | Adult psychiatric outpatients |
| Abiodun, 1993 [2] | CMD | GHQ-12 | Nigeria | Adult PCC attendees |
| Abiodun, 1994 [3] | CMDs, Anxiety disorders, Depressive disorders | GHQ-12,HADS-A, HADS-D | Nigeria | Adult hospital patients; Antenatal women |
| Abiodun, 2006 [4] | Postnatal depression | EPDS | Nigeria | Postnatal women |
| Adewuya et al., 2005 [5] | Postnatal depression, Postnatal major depressive episode | BDI, EPDS | Nigeria | Postnatal women |
| Adewuya et al., 2006a [6] | Depressive disorders, Major depressive episode | PHQ-9 | Nigeria | University students |
| Adewuya et al., 2006b [7] | Antenatal depression, Antenatal major depressive episode | EPDS | Nigeria | Antenatal women |
| Adewuya et al., 2007 [8] | Major depressive episode | BDI | Nigeria | Aged 13-18 students |
| Agoub et al., 2005 [9] | Postnatal depression | EPDS | Morocco | Postnatal women |
| Aguilar-Navarro et al., 2007 [10] | Depressive disorders | ENASEM | Mexico | Aged 65+ general population |
| Akena et al., 2012a [11] | Major depressive episode | AVIDI, AVIDI-4 | Uganda | Adults with HIV |
| Akena et al., 2013 [12] | Major depressive episode | CES-D, K-10, K-6, PHQ-9, PHQ-2 | Uganda | Adults with HIV |
| Ali et al., 2013 [13] | Major depressive episode | BDI | India | Adults with diabetes |
| Araya et al., 1992 [14] | CMDs | GHQ-12, CGHQ-12, SRQ-20 | Chile | Adult PCC attendees |
| Arunpongpaisal et al., 2009 [15] | Depressive disorders, Dysthemia, Major depressive episode | 2 question screen | Thailand | Adult general population |
| Aydin and Ulusahin, 2001 [16] | CMDs | GHQ-12 | Turkey | Adults with COPD; Adults with TB |
| Aydin et al., 2004 [17] | Postnatal depression | EPDS | Turkey | Postnatal women |
| Baggaley et al., 2007 [18] | Postnatal depression | K-10, K-6 | Burkina Faso | Postnatal women |
| Barua and Kar, 2010 [19] | Major depressive episode | WHO-5 | India | Aged 60+ general population |
| Basker et al., 2007 [20] | Depressive disorders | BDI | India | Aged16-17 PCC attendees |
| Basker et al., 2010 [21] | Depressive disorders | CDRS-R | India | Aged16-17 PCC attendees |
| Basoglu et al., 2001 [22] | Major depressive episode | TSSC | Turkey | Adult earthquake survivors |
| Betancourt et al., 2012 [23] | Major depressive episode | CES-DC | Rwanda | Aged 10-17 general population |
| Bhagwanjee et al., 1998 [24] | CMDs | SRQ-20 | South Africa | Adult general population |
| Biswas et al., 2009 [25] | CMDs | 2 question screen | India | Aged 60+ general population |
| Botega et al., 1995 [26] | Anxiety disorders, Depressive disorders | HADS-A, HADS-D | Brazil | Adult hospital inpatients |
| Botega et al., 1998 [27] | Anxiety disorders, Depressive disorders | HADS-A, HADS-D | Brazil | Adults with epilepsy |
| Brancaglion et al., 2013 [28] | Antental depression | EPDS | Brazil | Antenatal women |
| Bressan et al., 1998 [29] | Depressive disorders, Major depressive episode | CDSS | Brazil | Adults with schizophrenia |
| Caiuby et al., 2012 [30] | PTSD | IES-R | Brazil | Adult urban/domestic violence survivors |
| Camacho et al., 2009 [31] | Major depressive episode | CES-D | Colombia | Aged 11-16 students |
| Campo et al., 2006 [32] | Major depressive episode | ZSDS-10 | Colombia | Adult general population |
| Campo-Arias et al., 2005 [33] | Major depressive episode | ZSDS | Colombia | Female university students |
| Campo-Arias et al., 2006 [34] | Major depressive episode | ZSDS | Colombia | Adult general population |
| Campo-Arias et al., 2007 [35] | Major depressive episode | CES-D | Colombia | Adult general population |
| Cantilino et al., 2007 [36] | Postnatal depression | PDSS | Brazil | Postnatal women |
| Castelo et al., 2010 [37] | Major depressive episode | GDS-30, GDS-15, GDS-10, GDS-4, GDS-1 | Brazil | Aged 60+ PCC attendees |
| Castro et al., 2006 [38] | Anxiety disorders, Depressive disorders | HADS-A, HADS-D | Brazil | Adults with chronic pain |
| Chaaya et al., 2008 [39] | Depressive disorders | GDS-15 | Lebanon | Aged 60+ general population |
| Chagas et al., 2010 [40] | Major depressive episode | GDS-15, ZSDS, ZSDS minus physical items | Brazil | Adults with Parkinson's disease |
| Chen et al., 2009 [41] | CMDs | SRQ-20 | China | Adult general population |
| Chen et al., 2010 [42] | Major depressive episode | PHQ-9, PHQ-2 | China | Aged 60+ PCC attendees |
| Chen et al., 2013 [43] | Major depressive episode | PHQ-9 | China | Adult PCC attendees |
| Chibanda et al., 2010 [44] | Postnatal depression | EPDS | Zimbabwe | Postnatal women |
| Chipimo and Fylkesnes, 2010 [45] | CMDs | GHQ-12, SRQ-20, SRQ-10 | Zambia | Adult PCC attendees |
| Chipimo and Fylkesnes, 2013 [46] | CMDs | SRQ-5 | Zambia | Adult PCC attendees |
| Chishinga et al., 2011 [47] | Major depressive episode | CES-D | Zambia | Adults with HIV; Adults with TB |
| Chowdhury et al., 2004 [48] | Depressive disorders | PHQ-9 | India | Adult PCC and hospital outpatients |
| Corapcioglu and Ozer, 2004 [49] | Panic disorder, Depressive disorders, Major depressive disorder | PHQ-r | Turkey | Adult PCC attendees |
| Costa et al., 2006 [50] | Depressive disorders | GDS-30, GHQ-12 | Brazil | Aged 75+ general population |
| de Oliveira et al., 2010 [51] | Major depressive episode | NDDI-E | Brazil | Adults with epilepsy |
| de Oliveira et al., 2011 [52] | Major depressive episode | NDDI-E | Brazil | Adults with epilepsy |
| D'El Rey and Matos, 2009 [53] | Social anxiety disorder | Mini-SPIN | Brazil | Adult general population |
| Díaz et al., 2005 [54] | Major depressive episode | ZSDS, ZSDS-15, ZSDS-11, ZSDS-10, ZSDS-9 | Colombia | University students |
| El-Missiry et al., 2012 [55] | Depressive disorders | CDI | Egypt | Aged 14-17 female students |
| Fabregas et al., 2012 [56] | Major depressive episode | BDI, BDI-PC, HADS-D | Brazil | Adults with chronic hepatitis C |
| Fawzi et al., 2012 [57] | Major depressive episode | MDI-A | Egypt | Adult psychiatric outpatients |
| Fernandes et al., 2011 [58] | Antenatal depression | EPDS, K-10 | India | Antenatal women |
| Figueira et al., 2009 [59] | Postnatal depression | EPDS | Brazil | Postnatal women |
| Furlanetto et al., 2005 [60] | Moderate to severe depression | BDI-SF | Brazil | Adult hospital inpatients |
| Ganguly et al., 2013 [61] | Depressive disorders | PHQ-9 | India | Aged 14-18 PCC attendees |
| Gausia et al., 2007 [62] | Postnatal depression | EPDS | Bangladesh | Postnatal women |
| Gelaye et al., 2013 [63] | Major depressive episode | PHQ-9 | Ethiopia | Adult hospital outpatients |
| Giang et al., 2006 [64] | CMDs | SRQ-20 | Vietnam | Adult general population; Adult hospital outpatients |
| Gomes-Oliveira et al., 2012 [65] | Major depressive episode | BDI-II | Brazil | Adult general population |
| Goncalves et al., 2008 [66] | CMDs | SRQ-20 | Brazil | Adult general population |
| Hanlon et al., 2008 [67] | CMDs | EPDS, SRQ-20 | Ethiopia | Perinatal women |
| Hollander et al., 2007 [68] | CMDs, Anxiety disorders, Depressive disorders, PTSD | HSCL-25, HTQ-R | Tajikistan | Adult psychiatric outpatients |
| Jirapramukpitak et al., 2009 [69] | Major depressive episode | Euro-D | Thailand | Aged 60+ psychiatric clinic attendees |
| John and Russell, 2007 [70] | PTSD | IES-8 | Sri Lanka | Aged 13+ tsunami survivors |
| Jordans et al., 2008 [71] | CMDs | CPDS | Burundi | Aged 10-15 conflict survivors |
| Kaaya et al., 2002 [72] | Major depressive episode | HSCL-25, HSCL-revised | Burundi | Antenatal women with HIV |
| Khamseh et al., 2011 [73] | Major depressive episode | CES-D, PHQ-9 | Iran | Adults with diabetes |
| Kohrt et al., 2011 [74] | PTSD, Depressive disorders | CPSS, DSRS | Nepal | Aged 11-14 general population |
| Kulathunga et al., 2010 [75] | Depressive disorders | GDS-S | Sri Lanka | Aged 55+ psychogeriatric clinic attendees |
| Lau et al., 2010 [76] | Postnatal depression | EPDS | China | Postnatal women |
| Lawrie et al., 1998 [77] | Postnatal depression, Postnatal major depressive episode | EPDS | South Africa | Postnatal women |
| Lee et al., 2006 [78] | CMDs | GHQ-12 | China | Adult general population |
| Li et al., 2011 [79] | Postnatal depression, Postnatal major depressive episode | C-PDSS | China | Postnatal women |
| Liu et al., 2007 [80] | PTSD | 7 symptom screen | China | Aged 7-15 natural disaster survivors |
| Liu et al., 2008 [81] | PTSD | 7 symptom screen | China | Adult natural disaster survivors |
| Lotrakul et al., 2008 [82] | Major depressive episode | PHQ-9 | Thailand | Adult PCC attendees |
| Ludermir and Lewis, 2005 [83] | CMDs | SRQ-20 | Brazil | Adult general population |
| Mahfoud et al., 2011 [84] | CMDs | AYMH | Lebanon | Aged 10-14 social services attendees |
| Mahmud et al., 2003 [85] | Postnatal depression | EPDS | Malaysia | Postnatal women |
| Malakouti et al., 2006 [86] | Major depressive episode | GDS-15 | Iran | Aged 59+ general population |
| Malakouti et al., 2007 [87] | CMDs | GHQ-28, GHQ-15 | Iran | Aged 59+ general population |
| Mari and Williams, 1985 [88] | CMDs | GHQ-12, SRQ-20 | Brazil | Adult PCC attendees |
| Mazhari and Nakhaee, 2007 [89] | Postnatal depression, Postnatal major depressive episode | EPDS | Iran | Postnatal women |
| Mbewe et al., 2013 [90] | CMDs | 10 question screen | Zambia | Adults with epilepsy |
| Muhwezi et al., 2007 [91] | Major depressive episode | 4 item SWB scale | Uganda | Adult PCC attendees |
| Mumford et al., 2005 [92] | CMDs, Depressive disorders | PADQ | Pakistan | Adult PCC and hospital in/outpatients |
| Myer et al., 2008 [93] | Major depressive episode, PTSD | CES-D, HTQ | South Africa | Adults with HIV |
| N Azah et al., 2005 [94] | Depressive disorders, Major depressive episode | PHQ-9 | Malaysia | Adult PCC attendees |
| Nakimuli-Mpungu et al., 2012 [95] | Major depressive episode | SRQ-20 | Uganda | Adults with HIV |
| Nhiwatiwa et al., 1998 [96] | CMDs | SSQ | Zimbabwe | Postnatal women |
| Odenwald et al., 2007 [97] | PTSD | PDS | Somalia | Adult ex-combatants |
| Oruc et al., 2008 [98] | Major depressive episode, PTSD | HSCL-25, HTQ | Bosnia and Herzegovina | Adult PCC attendees |
| Osorio et al., 2007 [99] | Social anxiety disorder | MINI-SPIN | Brazil | University students |
| Osorio et al., 2010a [100] | Social anxiety disorder | BSPS | Brazil | University students |
| Osorio et al., 2010b [101] | Social anxiety disorder | SPIN | Brazil | University students |
| Osorio et al., 2011 [102] | Social anxiety disorder | BAI | Brazil | University students |
| Osorio et al., 2012 [103] | Depressive disorders | PHQ-9, PHQ-2 | Brazil | Adult hospital inpatients |
| Ozalp et al., 2008 [104] | Major depressive episode | HADS, HADS-D | Turkey | Adult women with breast cancer |
| Paradela et al., 2005 [105] | Depressive disorders | GDS-15 | Brazil | Aged 65+ PCC attendees |
| Patel et al., 1997 [106] | CMDs | SSQ | Zimbabwe | Adult PCC attendees |
| Patel et al., 2008 [107] | CMDs | GHQ-12, K-10, K-6, PHQ-9, SRQ-20 | India | Adult PCC attendees |
| Pence et al., 2012 [108] | Major depressive episode | PHQ-9 | Cameroon | Adults with HIV |
| Pineda et al., 2002 [109] | PTSD | PTSD-Checklist | Colombia | Adult guerilla attack survivors |
| Pinho et al., 2010 [110] | Depressive disorders, Major depressive episode | GDS-15 | Brazil | Aged 65+ with CAD |
| Pitanupong et al., 2007 [111] | Postnatal depression | EPDS | Thailand | Postnatal women |
| Pollock et al., 2006 [112] | Depressive disorders | EPDS, SRQ-20 | Mongolia | General population women of childbearing age |
| Puertas et al., 2004 [113] | CMDs | FACES, GHQ-5 | India | Adult hospital outpatients |
| Pupo et al., 2011 [114] | PTSD | CAPS | Brazil | Adult urban violence survivors |
| Regmi et al., 2002 [115] | Postnatal depression | EPDS | Nepal | Postnatal women |
| Rochat et al., 2013 [116] | Antental depression | EPDS, EPDS-7, EPDS-5, EPDS-3 | South Africa | Antenatal women |
| Rosliwati et al., 2008 [117] | Major depressive episode | CDI | Malaysia | Aged 7-17 hospital outpatients |
| Rueda-Jaimes et al., 2009 [118] | Major depressive episode | CES-D Brief, CES-D Ultra short | Colombia | Aged 13-17 students |
| Rumble et al., 1996 [119] | CMDs | SRQ | South Africa | Adult general population |
| Russell et al., 2012 [120] | Depressive disorders | BDI, CDRS-R | India | Aged 14-17 PCC attendees |
| Saipanish et al., 2009 [121] | Major depressive episode | WHO-5-T | Thailand | Adult PCC attendees |
| Salinas-Rodriguez et al., 2013 [122] | Depressive disorders | CESD-7 | Mexico | Adult general population |
| Salle et al., 2012 [123] | Major depressive episode | BDI, CES-D, CRS | Brazil | Aged 15-17 students |
| Santos et al., 2007 [124] | Postnatal depression | EPDS, SRQ-20 | Brazil | Postnatal women |
| Santos et al., 2013 [125] | Major depressive episode | PHQ-9 | Brazil | Adult general population |
| Scazufca et al., 2009 [126] | CMDs | SRQ-20 | Brazil | Aged 65+ general population |
| Segopolo et al., 2009 [127] | CMDs | GHQ-28 | Botswana | Adult PCC attendees |
| Shamasunder et al., 1986 [128] | CMDs | GHQ-5 | India | Adult relatives of psychiatric patients |
| Sheng, 2010 [129] | CMDs, Anxiety disorders, Depressive disorders | HADS, HADS-A, HADS-D, Case description | China | Adult psychiatric outpatients |
| Sherina et al., 2011 [130] | Depressive disorders | TQWHQ | Malaysia | Adult female PCC attendees |
| Sherina et al., 2012a [131] | Depressive disorders | PHQ-9 | Malaysia | Adult female PCC attendees |
| Sherina et al., 2012b [132] | Generalised anxiety disorder | GAD-7 | Malaysia | Adult female PCC attendees |
| Sibai et al., 2009 [133] | Depressive disorders | WHO-5-A | Lebanon | Aged 60+ general population |
| Spies et al., 2009a [134] | Panic disorder, SAD, Major depressive episode, PTSD | K-10 | South Africa | Antenatal women |
| Spies et al., 2009b [135] | GAD, Panic disorder, SAD, Major depressive disorder, PTSD | K-10 | South Africa | Adults with HIV |
| Stewart et al., 2009 [136] | Postnatal depression, Postnatal major depressive episode | SRQ-20 | Malawi | Postnatal women |
| Suttajit et al., 2013 [137] | Major depressive episode | CDSS | Thailand | Adults with schizophrenia |
| Tan et al., 2013 [138] | Depressive disorders | CDI | Malaysia | Aged 7-17 general population |
| Tesfaye et al., 2010 [139] | CMDs, Depressive disorders | EPDS, K-10, K-6 | Ethiopia | Postnatal women |
| Tran et al., 2012 [140] | CMDs | EPDS, GHQ-12, ZSAS | Vietnam | Postnatal men |
| Tran et al., 2013 [141] | CMDs, Anxiety disorders, Depressive disorders | DASS, DASS-A, DASS-D | Vietnam | General population women with young children |
| Tuan et al., 2004 [142] | CMDs | SRQ-20 | Vietnam | Adult female general population |
| Uwakwe, 2003 [143] | Postnatal depression | EPDS | Nigeria | Postnatal women |
| Vega-Dienstmaier et al., 2002 [144] | Postnatal major depressive episode | EPDS | Peru | Postnatal women |
| Vittayanont et al., 2006 [145] | Postnatal depression, Postnatal major depressive episode | PDSS | Thailand | Postnatal women |
| Wang et al., 2009 [146] | Antental depression | EPDS | China | Antenatal women |
| Weobong et al., 2009 [147] | CMDs | EPDS, PHQ-9, SRQ-20 | Ghana | Postnatal women |
| Wulsin et al., 2002 [148] | Major depressive episode | PHQ-9 | Honduras | Adult PCC attendee mothers |
| Xiao et al., 2009 [149] | Any depresive disorder | CDSS-C | China | Adults with schizophrenia |
| Yang et al., 2014 [150] | Generalised anxiety disorder, Major depressive disorder | HADS-A, HADS-D | China | Adult psycho-cardiological outpatients |
| Zhang et al., 2013 [151] | Major depressive episode | PHQ-9, PHQ-2 | China | University students |
| Zhou et al., 2013 [152] | Depressive disorders | HAM-D, PHQ-2 | China | Adults recovering from stroke |
| Zubaran et al., 2009 [153] | Postnatal depression | PDSS | Brazil | Postnatal women |

1. Abeyasinghe DRR, Tennakoon S, Rajapakse TN. The development and validation of the Peradeniya Depression Scale (PDS) - a culturally relevant tool for screening of depression in Sri Lanka. Journal of Affective Disorders. 2012;142(1/3):143-9. doi: <http://dx.doi.org/10.1016/j.jad.2012.04.019>.

2. Abiodun O. A study of mental morbidity among primary care patients in Nigeria. Comprehensive Psychiatry. 1993;34(1):10-3. doi: <http://dx.doi.org/10.1016/0010-440X%2893%2990030-8>. PubMed PMID: Peer Reviewed Journal: 1993-34811-001.

3. Abiodun OA. A validity study of the Hospital Anxiety and Depression Scale in general hospital units and a community sample in Nigeria. British Journal of Psychiatry. 1994;165(5):669-72. PubMed PMID: 7866683.

4. Abiodun O. Postnatal depression in primary care populations in Nigeria. General Hospital Psychiatry. 2006;28(2):133-6. doi: <http://dx.doi.org/10.1016/j.genhosppsych.2005.11.002>. PubMed PMID: Peer Reviewed Journal: 2006-03096-008.

5. Adewuya AO, Eegunranti AB, Lawal AM. Prevalence of postnatal depression in Western Nigerian women: A controlled study. International Journal of Psychiatry in Clinical Practice. 2005;9(1):60-4. doi: <http://dx.doi.org/10.1080/13651500510018211>. PubMed PMID: Peer Reviewed Journal: 2005-02066-011.

6. Adewuya AO, Ola BA, Afolabi OO. Validity of the patient health questionnaire (PHQ-9) as a screening tool for depression amongst Nigerian university students. [References]. 2006. doi: <http://dx.doi.org/10.1016/j.jad.2006.05.021>. PubMed PMID: Peer Reviewed Journal: 2006-20190-011.

7. Adewuya AO, Ola BA, Dada AO, Fasoto OO. Validation of the Edinburgh Postnatal Depression Scale as a screening tool for depression in late pregnancy among Nigerian women. Journal of Psychosomatic Obstetrics & Gynecology. 2006;27(4):267-72. doi: <http://dx.doi.org/10.1080/01674820600915478>. PubMed PMID: Peer Reviewed Journal: 2007-01540-010.

8. Adewuya AO, Ola BA, Aloba OO. Prevalence of major depressive disorders and a validation of the Beck Depression Inventory among Nigerian adolescents. European Child & Adolescent Psychiatry. 2007;16(5):287-92. doi: <http://dx.doi.org/10.1007/s00787-006-0557-0>. PubMed PMID: Peer Reviewed Journal: 2007-13379-001.

9. Agoub M, Moussaoui D, Battas O. Prevalence of postpartum depression in a Moroccan sample. Archives of Women's Mental Health. 2005;8(1):37-43. doi: <http://dx.doi.org/10.1007/s00737-005-0069-9>. PubMed PMID: Peer Reviewed Journal: 2005-06339-006.

10. Aguilar-Navarro SG, Fuentes-Cantu A, Avila-Funes JA, Garcia-Mayo EJ. Validity and reliability of the screening questionnaire for geriatric depression used in the Mexican Health and Age Study. [Spanish]. Salud Publica de Mexico. 2007;49(4):256-62.

11. Akena D, Joska J, Musisi S, Stein DJ. Sensitivity and specificity of a visual depression screening instrument among HIV-positive individuals in Uganda, an area with low literacy. AIDS & Behavior. 2012;16(8):2399-406. doi: <http://dx.doi.org/10.1007/s10461-012-0267-1>.

12. Akena D, Joska J, Obuku EA, Stein DJ. Sensitivity and specificity of clinician administered screening instruments in detecting depression among HIV-positive individuals in Uganda. AIDS Care. 2013;25(10):1245-52. doi: <http://dx.doi.org/10.1080/09540121.2013.764385>.

13. Ali N, Jyotsna VP, Nand K, Kalaivani M. Prevalence of depression among type 2 diabetes compared to healthy non diabetic controls. Journal of the Association of Physicians of India. 2013;61(September):31-3.

14. Araya RI, Wynn R, Lewis G. Comparison of two self administered psychiatric questionnaires (GHQ-12 and SRQ-20) in primary care in Chile. Social Psychiatry and Psychiatric Epidemiology. 1992;27(4):168-73. PubMed PMID: 1992283278.

15. Arunpongpaisal S, Kongsuk T, Maneethorn N, Maneethorn B, Wannasawek K, Leejongpermpoon J, et al. Development and validity of two-question screening test for depressive disorders in Northeastern Thai community. Asian Journal of Psychiatry. 2009;2(4):149-52. doi: <http://dx.doi.org/10.1016/j.ajp.2009.10.002>. PubMed PMID: 2009612897.

16. Aydin IO, Ulusahin A. Depression, anxiety comorbidity, and disability in tuberculosis and chronic obstructive pulmonary disease patients: applicability of GHQ-12. General Hospital Psychiatry. 2001;23(2):77-83.

17. Aydin N, Inandi T, Yigit A, Nalan Sahin Hodoglugil N. Validation of the Turkish version of the Edinburgh Postnatal Depression Scale among women within their first postpartum year. Social Psychiatry and Psychiatric Epidemiology. 2004;39(6):483-6. doi: <http://dx.doi.org/10.1007/s00127-004-0770-4>. PubMed PMID: 2004444881.

18. Baggaley RF, Ganaba R, Filippi V, Kere M, Marshall T, Sombie I, et al. Detecting depression after pregnancy: The validity of the K10 and K6 in Burkina Faso. Tropical Medicine and International Health. 2007;12(10):1225-9. doi: <http://dx.doi.org/10.1111/j.1365-3156.2007.01906.x>. PubMed PMID: 2007516494.

19. Barua A, Kar N. Screening for depression in elderly Indian population. Indian Journal of Psychiatry. 2010;52(2):150-3.

20. Basker M, Moses PD, Russell S, Russell PSS. The psychometric properties of beck depression Inventory for adolescent depression in a primary-care paediatric setting in India. Child and Adolescent Psychiatry and Mental Health. 2007;1(8). doi: <http://dx.doi.org/10.1186/1753-2000-1-8>. PubMed PMID: 2008285046.

21. Basker M, Russell PSS, Russell S, Moses P. Validation of the children's depression rating scale-revised for adolescents in primary-care pediatric use in India. Indian Journal of Medical Sciences. 2010;64(2):72-80. doi: <http://dx.doi.org/10.4103/0019-5359.94403>. PubMed PMID: 2012218309.

22. Basoglu M, Salcioglu E, Livanou M, Ozeren M, Aker T, Kilic C, et al. A study of the validity of a screening instrument for traumatic stress in earthquake survivors in Turkey. 2001. doi: <http://dx.doi.org/10.1023/A:1011156505957>. PubMed PMID: Peer Reviewed Journal: 2002-10010-006.

23. Betancourt T, Scorza P, Meyers-Ohki S, Mushashi C, Kayiteshonga Y, Binagwaho A, et al. Validating the center for epidemiological studies depression scale for children in Rwanda. Journal of the American Academy of Child and Adolescent Psychiatry. 2012;51(12):1284-92. doi: <http://dx.doi.org/10.1016/j.jaac.2012.09.003>. PubMed PMID: 2012704370.

24. Bhagwanjee A, Parekh A, Paruk Z, Petersen I, Subedar H. Prevalence of minor psychiatric disorders in an adult African rural community in South Africa. Psychological Medicine. 1998;28:1137-47.

25. Biswas SS, Gupta R, Vanjare HA, Bose S, Patel JA, Selvarajan S, et al. Depression in the elderly in Vellore, South India: The use of a two-question screen. International Psychogeriatrics. 2009;21(2):369-71. doi: <http://dx.doi.org/10.1017/S1041610208008259>. PubMed PMID: 2009471860.

26. Botega NJ, Bio MR, Zomignani MA, Garcia Jr C, Pereira WA. Mood disorders among inpatients in ambulatory and validation of the anxiety and depression scale HAD. [Portuguese] Transtornos do humor em enfermaria de clinica medica e validacao de escala de medida (HAD) de ansiedade e depressao. Revista de saude publica. 1995;29(5):355-63. PubMed PMID: 8731275.

27. Botega NJ, Ponde MP, Medeiros P, Lima MG, Guerreiro CAM. Validation of the Hospital Anxiety and Depression Scale in ambulatory epileptic patients. Jornal Brasileiro de Psiquiatria. 1998;47(6):285-9. PubMed PMID: Peer Reviewed Journal: 1998-10557-001.

28. Brancaglion MY, Couto TCe, Vasconcellos AG, Malloy-Diniz LF, Nicolato R, Correa H. Edinburgh Postnatal Depression Scale for screening antepartum depression in the Brazilian public health system: Clinical Neuropsychiatry: Journal of Treatment Evaluation. Vol.10(2), Apr 2013, pp. 102-106.; 2013.

29. Bressan RA, Chaves AC, Shirakawa I, Mari JDJ. Validity study of the Brazilian version of the Calgary Depression Scale for Schizophrenia. Schizophrenia Research. 1998;32(1):41-9. doi: <http://dx.doi.org/10.1016/S0920-9964%2898%2900039-5>. PubMed PMID: 1998245641.

30. Caiuby AVS, Lacerda SS, Quintana MI, Torii TS, Andreoli SB. Cross-cultural adaptation of the Brazilian version of the impact of Events Scale-Revised (IES-R)

Adaptacao transcultural da versao brasileira da Escala do Impacto do Evento - Revisada (IES-R). Cadernos de Saude Publica. 2012;28(3):597-603. doi: <http://dx.doi.org/10.1590/S0102-311X2012000300019>. PubMed PMID: 22415191.

31. Camacho PA, Rueda-Jaimes GE, Latorre JF, Navarro-Mancilla AA, Escobar M, Franco JA. Validity and reliability of the Center for Epidemiologic Studies-Depression scale in Colombian adolescent students. [Spanish]

Validez y confiabilidad de la escala del Center for Epidemiologic Studies-Depression en estudiantes adolescentes de Colombia. Biomedica : revista del Instituto Nacional de Salud. 2009;29(2):260-9. PubMed PMID: 20128351.

32. Campo A, Diaz LA, Rueda GE. Validity of the brief Zung's scale for screening major depressive episode among the general population from Bucaramanga, Colombia. [Spanish] Validez de la escala breve de Zung para tamizaje del episodio depresivo mayor en la poblacion general de Bucaramanga, Colombia. Biomedica : revista del Instituto Nacional de Salud. 2006;26(3):415-23. PubMed PMID: 17176005.

33. Campo-Arias A, Diaz-Martinez LA, Rueda-Jaimes GE, Barros-Bermudez JA. Validation of Zung's Self-Rating Depression Scale Among University Student Women from Bucaramanga, Colombia. [Spanish]. [References]: Revista Colombiana de Psiquiatria. Vol.34(1[41]; 41), Mar 2005, pp. 54-62.; 2005.

34. Campo-Arias A, Diaz-Martinez LA, Rueda-Jaimes GE, del Pilar Cadena L, Hernandez NL. Validation of Zung's self-rating depression scale among the Colombian general population. Social Behavior and Personality. 2006;34(1):87-94. doi: <http://dx.doi.org/10.2224/sbp.2006.34.1.87>. PubMed PMID: Peer Reviewed Journal: 2006-02835-008.

35. Campo-Arias A, Diaz-Martinez LA, Rueda-Jaimes GE, del Pilar Cadena-Afanador L, Hernandez NL. Psychometric properties of the CES-D Scale among Colombian adults from the general population. Revista Colombiana de Psiquiatria. 2007;36(4):664-74. PubMed PMID: Peer Reviewed Journal: 2008-01507-005.

36. Cantilino A, Carvalho AJ, Maia A, Albuquerque C, Cantilino G, Sougey EB. Translation, validation and cultural aspects of postpartum depression screening scale in Brazilian Portuguese. Transcultural Psychiatry. 2007;44(4):672-84. doi: <http://dx.doi.org/10.1177/1363461507083904>. PubMed PMID: 2007609691.

37. Castelo MS, Coelho-Filho JM, Carvalho AF, Lima JWO, Noleto JCS, Ribeiro KG, et al. Validity of the Brazilian version of the Geriatric Depression Scale (GDS) among primary care patients. International Psychogeriatrics. 2010;22(1):109-13. doi: <http://dx.doi.org/10.1017/S1041610209991219>. PubMed PMID: 2010065436.

38. Castro MMC, Quarantini L, Batista-Neves S, Kraychete DC, Daltro C, Miranda-Scippa A. Validity of the hospital anxiety and depression scale in patients with chronic pain. [Portuguese] Validade da escala hospitalar de ansiedade e depressao em pacientes com dor cronica. Revista Brasileira de Anestesiologia. 2006;56(5):470-7. PubMed PMID: 2006496960.

39. Chaaya M, Sibai A-M, El Roueiheb Z, Chemaitelly H, Chahine LM, Al-Amin H, et al. Validation of the Arabic version of the short Geriatric Depression Scale (GDS-15). International Psychogeriatrics. 2008;20(3):571-81.

40. Chagas MHN, Tumas V, Loureiro SR, Hallak JEC, Trzesniak C, de Sousa JPM, et al. Validity of a Brazilian version of the Zung self-rating depression scale for screening of depression in patients with Parkinson's disease. Parkinsonism and Related Disorders. 2010;16(1):42-5. doi: <http://dx.doi.org/10.1016/j.parkreldis.2009.07.010>. PubMed PMID: 2010045419.

41. Chen S, Zhao G, Li L, Wang Y, Chiu H, Caine E. Psychometric properties of the Chinese version of the Self-Reporting Questionnaire 20 (SRQ-20) in community settings. International Journal of Social Psychiatry. 2009;55(6):538-47. doi: <http://dx.doi.org/10.1177/0020764008095116>. PubMed PMID: Peer Reviewed Journal: 2009-20721-005.

42. Chen S, Chiu H, Xu B, Ma Y, Jin T, Wu M, et al. Reliability and validity of the PHQ-9 for screening late-life depression in Chinese primary care. International Journal of Geriatric Psychiatry. 2010;25(11):1127-33. doi: <http://dx.doi.org/10.1002/gps.2442>. PubMed PMID: 2010625787.

43. Chen S, Fang Y, Chiu H, Fan H, Jin T, Conwell Y. Validation of the nine-item Patient Health Questionnaire to screen for major depression in a Chinese primary care population. Asia-Pacific Psychiatry. 2013;5(2):61-8. doi: <http://dx.doi.org/10.1111/appy.12063>.

44. Chibanda D, Mangezi W, Tshimanga M, Woelk G, Rusakaniko P, Stranix-Chibanda L, et al. Validation of the Edinburgh Postnatal Depression Scale among women in a high HIV prevalence area in urban Zimbabwe. Archives of Women's Mental Health. 2010;13(3):201-6. doi: <http://dx.doi.org/10.1007/s00737-009-0073-6>. PubMed PMID: Peer Reviewed Journal: 2010-10374-004.

45. Chipimo PJ, Fylkesnes K. Comparative validity of screening instruments for mental distress in Zambia. Clinical Practice and Epidemiology in Mental Health. 2010;6:4-15. doi: <http://dx.doi.org/10.2174/1745017901006010004>. PubMed PMID: Peer Reviewed Journal: 2011-17157-001.

46. Chipimo PJ, Fylkesnes K. Case-finding for mental distress in primary health care: An evaluation of the performance of a five-item screening instrument. Health. 2013;5(3a):627-36.

47. Chishinga N, Kinyanda E, Patel V, Seedat S. Validation of brief screening tools for depressive and alcohol use disorders among TB and HIV patients in primary care in Zambia. BMC Psychiatry. 2011;11(75).

48. Chowdhury AN, Sayanti G, Debasish S. Bengali adaptation of brief patient health questionnaire for screening depression at primary care. Journal of the Indian Medical Association. 2004;102(10):544-7.

49. Corapcioglu A, Ozer GU. Adaptation of revised Brief PHQ (Brief-PHQ-r) for diagnosis of depression, panic disorder and somatoform disorder in primary healthcare settings. International Journal of Psychiatry in Clinical Practice. 2004;8(1):11-8. PubMed PMID: 2004122051.

50. Costa E, Barreto SM, Uchoa E, Firmo JOA, Lima-Costa MF, Prince M. Is the GDS-30 better than the GHQ-12 for screening depression in elderly people in the community? The Bambui Health Aging Study (BHAS). International Psychogeriatrics. 2006;18(3):493-503. doi: <http://dx.doi.org/10.1017/S1041610205002954>. PubMed PMID: 2006359733.

51. de Oliveira GN, Kummer A, Salgado JV, Portela EJ, Sousa-Pereira SR, David AS, et al. Brazilian version of the Neurological Disorders Depression Inventory for Epilepsy (NDDI-E). Epilepsy & Behavior. 2010;19(3):328-31. doi: <http://dx.doi.org/10.1016/j.yebeh.2010.07.013>.

52. de Oliveira GNM, de Araujo Filho GM, Kummer A, Salgado JV, Portela EJ, Sousa-Pereira SR, et al. Neurological disorders depression inventory for epilepsy (NDDI-E): Brazilian version of a screening instrument. [Portuguese]

Inventario de depressao em transtornos neurologicos para a epilepsia (IDTN-E): Versao Brasileira de um instrumento de rastreamento. Journal of Epilepsy and Clinical Neurophysiology. 2011;17(2):49-53. doi: <http://dx.doi.org/10.1590/S1676-26492011000200004>. PubMed PMID: 2011612583.

53. D'El Rey GJF, Matos CW. Validation of the Portuguese version of the Mini-Social Phobia Inventory (Mini-SPIN). [Portuguese] Promocao a saude e vigilancia de violencias: efetividade e perspectivas. Ciencia & Saude Coletiva. 2009;14(5):1681-6. doi: <http://dx.doi.org/10.1590/S1413-81232009000500009>.

54. Díaz LA, Campo A, Rueda GE, Barros JA. Propuesta de una versión abreviada de la escala de Zung para depresión. Colombia Médica. 2005;36(3):168-72.

55. El-Missiry A, Soltan M, Abdel Hadi M, Sabry W. Screening for depression in a sample of Egyptian secondary school female students. Journal of Affective Disorders. 2012;136(1-2). doi: <http://dx.doi.org/10.1016/j.jad.2011.06.031>.

56. Fabregas BC, Vitorino FD, Rocha DM, Moura AS, Carmo RA, Teixeira AL. Screening inventories to detect depression in chronic hepatitis C patients. General Hospital Psychiatry. 2012;34(1):40-5. doi: <http://dx.doi.org/10.1016/j.genhosppsych.2011.09.002>.

57. Fawzi MH, Fawzi MM, Abu-Hindi W. Arabic version of the Major Depression Inventory as a diagnostic tool: Reliability and concurrent and discriminant validity. Eastern Mediterranean Health Journal. 2012;18(4):304-10. PubMed PMID: 2012508161.

58. Fernandes MC, Srinivasan K, Stein AL, Menezes G, Sumithra RS, Ramchandani PG. Assessing prenatal depression in the rural developing world: A comparison of two screening measures. Archives of Women's Mental Health. 2011;14(3):209-16. doi: <http://dx.doi.org/10.1007/s00737-010-0190-2>. PubMed PMID: 2011449287.

59. Figueira P, Correa H, Malloy-Diniz L, Romano-Silva MA. Edinburgh Postnatal Depression Scale for screening in the public health system. [Portuguese] Escala de Depressão Pós-natal de Edimburgo para triagem no sistema público de saúde. Revista de Saude Publica. 2009;43(Suppl.):79-84. doi: <http://dx.doi.org/10.1590/S0034-89102009000800012>.

60. Furlanetto LM, Mendlowicz MV, Bueno JR. The validity of the Beck Depression Inventory-Short Form as a screening and diagnostic instrument for moderate and severe depression in medical inpatients. Journal of Affective Disorders. 2005;86:87-91.

61. Ganguly S, Samanta M, Roy P, Chatterjee S, Kaplan DW, Basu B. Patient health questionnaire-9 as an effective tool for screening of depression among Indian adolescents. Journal of Adolescent Health. 2013;52(5):546-51. doi: <http://dx.doi.org/10.1016/j.jadohealth.2012.09.012>.

62. Gausia K, Fisher C, Algin S, Oosthuizen J. Validation of the Bangla version of the Edinburgh Postnatal Depression Scale for a Bangladeshi sample. Journal of Reproductive and Infant Psychology. 2007;25(4):308-15. doi: <http://dx.doi.org/10.1080/02646830701644896>. PubMed PMID: 2007575377.

63. Gelaye B, Williams MA, Lemma S, Deyessa N, Bahretibeb Y, Shibre T, et al. Validity of the patient health questionnaire-9 for depression screening and diagnosis in East Africa. Psychiatry Research. 2013;210(2):653-61. doi: <http://dx.doi.org/10.1016/j.psychres.2013.07.015>.

64. Giang BK, Allebeck P, Kullgren G, Van Tuan N. The Vietnamese version of the Self Reporting Questionnaire 20 (SRQ-20) in detecting mental disorders in rural Vietnam: A validation study. International Journal of Social Psychiatry. 2006;52(2):175-84. doi: <http://dx.doi.org/10.1177/0020764006061251>. PubMed PMID: 2006161161.

65. Gomes-Oliveira MH, Gorenstein C, Lotufo Neto F, Andrade LH, Wang YP. Validation of the Brazilian Portuguese version of the Beck Depression Inventory-II in a community sample. Revista Brasileira de Psiquiatria. 2012;34(4):389-94.

66. Goncalves DM, Stein AT, Kapczinski F. Performance of the Self-Reporting Questionnaire as a psychiatric screening questionnaire: A comparative study with Structured Clinical Interview for DSM-IV-TR. [Portuguese] Avaliacao de desempenho do Self-Reporting Questionnaire como instrumento de rastreamento psiquiatrico: Um estudo comparativo com o Structured Clinical Interview for DSM-IV-TR. Cadernos de Saude Publica. 2008;24(2):380-90. doi: <http://dx.doi.org/10.1590/S0102-311X2008000200017>. PubMed PMID: 18278285.

67. Hanlon C, Girmay M, Atalay A, Mesfin A, Abdulreshid A, Hughes M, et al. Detecting perinatal common mental disorders in Ethiopia: validation of the self-reporting questionnaire and Edinburgh Postnatal Depression Scale. Journal of Affective Disorders. 2008;108(3):251-62. doi: <http://dx.doi.org/10.1016/j.jad.2007.10.023>.

68. Hollander AC, Ekblad S, Mukhamadiev D, Muminova R. The validity of screening instruments for posttraumatic stress disorder, depression, and other anxiety symptoms in Tajikistan. Journal of Nervous and Mental Disease. 2007;195(11):955-8. doi: <http://dx.doi.org/10.1097/NMD.0b013e318159604b>. PubMed PMID: 2007560650.

69. Jirapramukpitak T, Darawuttimaprakorn N, Punpuing S, Abas M. Validation and factor structure of the Thai version of the EURO-D scale for depression among older psychiatric patients. Aging & Mental Health. 2009;13(6):899-904. doi: <http://dx.doi.org/10.1080/13607860903046479>. PubMed PMID: Peer Reviewed Journal: 2009-22180-013.

70. John PB, Russell PSS. Validation of a measure to assess Post-Traumatic Stress Disorder: A Sinhalese version of impact of event scale. Clinical Practice and Epidemiology in Mental Health. 2007;3(4). doi: <http://dx.doi.org/10.1186/1745-0179-3-4>. PubMed PMID: 2007116276.

71. Jordans MJ, Komproe IH, Ventevogel P, Tol WA, de Jong JT. Development and validation of the child psychosocial distress screener in Burundi. American Journal of Orthopsychiatry. 2008;78(3):290-9. doi: <http://dx.doi.org/10.1037/a0014216>.

72. Kaaya S, Fawzi M, Mbwambo J, Lee B, Msamanga G, Fawzi W. Validity of the Hopkins Symptom Checklist-25 amongst HIV-positive pregnant women in Tanzania. Acta Psychiatr Scand. 2002;106:9–19.

73. Khamseh ME, Baradaran HR, Javanbakht A, Mirghorbani M, Yadollahi Z, Malek M. Comparison of the CES-D and PHQ-9 depression scales in people with type 2 diabetes in Tehran, Iran. BMC Psychiatry. 2011;11. doi: <http://dx.doi.org/10.1186/1471-244X-11-61>. PubMed PMID: Peer Reviewed Journal: 2011-12954-001.

74. Kohrt BA, Jordans MJD, Tol WA, Luitel NP, Maharjan SM, Upadhaya N. Validation of cross-cultural child mental health and psychosocial research instruments: Adapting the Depression Self-Rating Scale and Child PTSD Symptom Scale in Nepal. BMC Psychiatry. 2011;11(127). doi: <http://dx.doi.org/10.1186/1471-244X-11-127>. PubMed PMID: 2011476653.

75. Kulathunga M, Umayal S, Somaratne S, Srikanth S, Kathriarachchi S, Krd DS. Validation of the Geriatric Depression Scale for an elderly Sri Lankan clinic population. Indian Journal of Psychiatry. 2010;52(3):254-6. doi: <http://dx.doi.org/10.4103/0019-5545.70979>. PubMed PMID: 2010578832.

76. Lau Y, Wang Y, Yin L, Chan KS, Guo X. Validation of the Mainland Chinese version of the Edinburgh Postnatal Depression Scale in Chengdu mothers. International Journal of Nursing Studies. 2010;47(9):1139-51. doi: <http://dx.doi.org/10.1016/j.ijnurstu.2010.02.005>. PubMed PMID: 20219196.

77. Lawrie TA, Hofmeyr GJ, De Jager M, Berk M. Validation of the Edinburgh Postnatal Depression Scale on a cohort of South African women. South African Medical Journal. 1998;88(10):1340-4. PubMed PMID: 1998364559.

78. Lee DT, Yip WC, Chen Y, Meng Q, Kleinman A. Ethno-psychometric evaluation of the General Health Questionnaire in rural China. Psychological Medicine. 2006;36(2):249-55.

79. Li L, Liu F, Zhang H, Wang L, Chen X. Chinese version of the Postpartum Depression Screening Scale: Translation and validation. Nursing Research. 2011;60(4):231-9. doi: <http://dx.doi.org/10.1097/NNR.0b013e3182227a72>. PubMed PMID: Peer Reviewed Journal: 2011-14248-004.

80. Liu A, Tan H, Zhou J, Li S, Yang T, Sun Z, et al. Brief screening instrument of posttraumatic stress disorder for children and adolescents 7-15 years of age. Child Psychiatry and Human Development. 2007;38(3):195-202. doi: <http://dx.doi.org/10.1007/s10578-007-0056-7>. PubMed PMID: Peer Reviewed Journal: 2008-00116-003.

81. Liu A, Tan H, Zhou J, Li S, Yang T, Tang X, et al. A short DSM-IV screening scale to detect posttraumatic stress disorder after a natural disaster in a Chinese population. Psychiatry Research. 2008;159(3):376-81. doi: <http://dx.doi.org/10.1016/j.psychres.2007.08.015>.

82. Lotrakul M, Sumrithe S, Saipanish R. Reliability and validity of the Thai version of the PHQ-9. BMC Psychiatry. 2008;8. doi: <http://dx.doi.org/10.1186/1471-244X-8-46>. PubMed PMID: Peer Reviewed Journal: 2008-10028-001.

83. Ludermir AB, Lewis G. Investigating the effect of demographic and socioeconomic variables on misclassification by the SRQ-20 compared with a psychiatric interview. Social Psychiatry and Psychiatric Epidemiology. 2005;40(1):36-41. doi: <http://dx.doi.org/10.1007/s00127-005-0840-2>. PubMed PMID: 2005020891.

84. Mahfoud Z, Abdulrahim S, Taha MB, Harpham T, El Hajj T, Makhoul J, et al. Validation of the Arab Youth Mental Health scale as a screening tool for depression/anxiety in Lebanese children. Child and Adolescent Psychiatry and Mental Health. 2011;5(9). doi: <http://dx.doi.org/10.1186/1753-2000-5-9>. PubMed PMID: 2011184603.

85. Mahmud WMRW, Awang A, Mohamed MN. Revalidation of the Malay version of the Edinburgh postnatal depression scale (EPDS) among Malay postnatal women attending the Bakar Bata Health Center in Alor Setar, Kedah, North West of Peninsular Malaysia. Malaysian Journal of Medical Sciences. 2003;10(2):71-5. PubMed PMID: 2004241672.

86. Malakouti SK, Fatollahi P, Mirabzadeh A, Salavati M, Zandi T. Reliability, validilty and factor structure of the GDS-15 in Iranian elderly. International Journal of Geriatric Psychiatry. 2006;21(6):588-93. doi: <http://dx.doi.org/10.1002/gps.1533>. PubMed PMID: 2006333512.

87. Malakouti SK, Fatollahi P, Mirabzadeh A, Zandi T. Reliability, validity and factor structure of the GHQ-28 used among elderly Iranians. International Psychogeriatrics. 2007;19(4):623-34. doi: <http://dx.doi.org/10.1017/S1041610206004522>. PubMed PMID: Peer Reviewed Journal: 2007-12047-002.

88. Mari JJ, Williams P. A comparison of the validity of two psychiatric screening questionnaires (GHQ-12 and SRQ-20) in Brazil, using Relative Operating Characteristic (ROC) analysis. Psychological Medicine. 1985;15(3):651-9. doi: <http://dx.doi.org/10.1017/S0033291700031500>. PubMed PMID: Peer Reviewed Journal: 1986-18964-001.

89. Mazhari S, Nakhaee N. Validation of the Edinburgh Postnatal Depression Scale in an Iranian sample. Archives of Women's Mental Health. 2007;10(6):293-7. doi: <http://dx.doi.org/10.1007/s00737-007-0204-x>. PubMed PMID: Peer Reviewed Journal: 2008-03099-007.

90. Mbewe EK, Uys LR, Nkwanyana NM, Birbeck GL. A primary healthcare screening tool to identify depression and anxiety disorders among people with epilepsy in Zambia. Epilepsy & Behavior. 2013;27(2):296-300. doi: <http://dx.doi.org/10.1016/j.yebeh.2013.01.025>.

91. Muhwezi WW, Agren H, Musisi S. Detection of major depression in Ugandan primary health care settings using simple questions from a subjective well-being (SWB) subscale. Soc Psychiatry Psychiatr Epidemiol. 2007;42:61-9.

92. Mumford DB, Ayub M, Karim R, Izhar N, Asif A, Bavington JT. Development and validation of a questionnaire for anxiety and depression in Pakistan. Journal of Affective Disorders. 2005;88:175–82.

93. Myer L, Smit J, Le Roux L, Parker S, Stein DJ, Seedat S. Common mental disorders among HIV-infected individuals in South Africa: Prevalence, predictors, and validation of brief psychiatric rating scales. AIDS Patient Care and STDs. 2008;22(2):147-58. doi: <http://dx.doi.org/10.1089/apc.2007.0102>. PubMed PMID: Peer Reviewed Journal: 2008-02117-004.

94. N Azah MN, M Shah ME, Juwita S, Bahri IS, Rushidi WMWM, Jamil YM. Validation of the Malay Version Brief Patient Health Questionnaire (PHQ-9) among Adult Attending Family Medicine Clinics. International Medical Journal. 2005;12(4):259-63. PubMed PMID: Peer Reviewed Journal: 2005-16625-003.

95. Nakimuli-Mpungu E, Mojtabai R, Alexandre PK, Katabira E, Musisi S, Nachega JB, et al. Cross-cultural adaptation and validation of the self-reporting questionnaire among HIV+ individuals in a rural ART program in southern Uganda. HIV/AIDS - Research and Palliative Care. 2012;4:51-60. doi: <http://dx.doi.org/10.2147/hiv.s29818>. PubMed PMID: 2012258267.

96. Nhiwatiwa S, Patel V, Acuda W. Predicting postnatal mental disorder with a screening questionnaire: A prospective cohort study from Zimbabwe. Journal of Epidemiology and Community Health. 1998;52(4):262-6. doi: <http://dx.doi.org/10.1136/jech.52.4.262>. PubMed PMID: Peer Reviewed Journal: 1998-01703-001.

97. Odenwald M, Lingenfelder B, Schauer M, Neuner F, Rockstroh B, Hinkel H, et al. Screening for Posttraumatic Stress Disorder among Somali ex-combatants: a validation study. Conflict and Health. 2007;1(10).

98. Oruc L, Kapetanovic A, Pojskic N, Miley K, Forstbauer S, Mollica RF, et al. Screening for PTSD and depression in Bosnia and Herzegovina: Validating the Harvard Trauma Questionnaire and the Hopkins Symptom Checklist. International Journal of Culture and Mental Health. 2008;1(2):105-16. doi: <http://dx.doi.org/10.1080/17542860802456620>. PubMed PMID: Peer Reviewed Journal: 2010-18388-003.

99. Osorio FdL, Crippa JA, Loureiro SR. A study of the discriminative validity of a screening tool (MINI-SPIN) for social anxiety disorder applied to Brazilian university students. European Psychiatry. 2007;22(4):239-43. doi: <http://dx.doi.org/10.1016/j.eurpsy.2007.01.003>. PubMed PMID: Peer Reviewed Journal: 2007-07746-006.

100. Osorio FL, Crippa JA, Loureiro SR. Study of the psychometric qualities of the Brief Social Phobia Scale (BSPS) in Brazilian university students. European Psychiatry: the Journal of the Association of European Psychiatrists. 2010;25(3):178-88. doi: <http://dx.doi.org/10.1016/j.eurpsy.2009.08.002>.

101. Osorio FL, Crippa JAS, Loureiro SR. Evaluation of the psychometric properties of the Social Phobia Inventory in university students. Comprehensive Psychiatry. 2010;51(6):630-40. doi: <http://dx.doi.org/10.1016/j.comppsych.2010.03.004>. PubMed PMID: Peer Reviewed Journal: 2011-08012-011.

102. Osorio FL, Crippa JAS, Loureiro SR. Further psychometric study of the Beck Anxiety Inventory including factorial analysis and social anxiety disorder screening. International Journal of Psychiatry in Clinical Practice. 2011;15(4):255-62. doi: <http://dx.doi.org/10.3109/13651501.2011.605955>. PubMed PMID: 2011563830.

103. Osorio F, Carvalho A, Fracalossi T, Crippa J, Loureiro E. Are two items sufficient to screen for depression within the hospital context. International Journal of Psychiatry in Medicine. 2012;44(2):141-8. doi: <http://dx.doi.org/10.2190/PM.44.2.e>. PubMed PMID: 2012726111.

104. Ozalp E, Soygur H, Cankurtaran E, Turhan L, Akbiyik D, Geyik P. Psychiatric morbidity and its screening in Turkish women with breast cancer: A comparison between the HADS and SCID tests. Psycho-Oncology. 2008;17(7):668-75. doi: <http://dx.doi.org/10.1002/pon.1286>. PubMed PMID: Peer Reviewed Journal: 2008-10809-004.

105. Paradela EMP, Lourenco RA, Veras RP. Validation of geriatric depression scale in a general outpatient clinic. Revista de Saude Publica. 2005;39(6):918-23. doi: <http://dx.doi.org/10.1590/S0034-89102005000600008>. PubMed PMID: 2006196452.

106. Patel V, Simunyu E, Gwanzura F, Lewis G, Mann A. The Shona Symptom Questionnaire: The development of an indigenous measure of common mental disorders in Harare. Acta Psychiatrica Scandinavica 1997;95(6):469-75. doi: <http://dx.doi.org/10.1111/j.1600-0447.1997.tb10134.x>. PubMed PMID: Peer Reviewed Journal: 1998-00164-003.

107. Patel V, Araya R, Chowdhary N, King M, Kirkwood B, Nayak S, et al. Detecting common mental disorders in primary care in India: a comparison of five screening questionnaires. Psychological Medicine. 2008;38:221-8.

108. Pence BW, Gaynes BN, Atashili J, O'Donnell JK, Tayong G, Kats D, et al. Validity of an interviewer-administered patient health questionnaire-9 to screen for depression in HIV-infected patients in Cameroon. Journal of Affective Disorders 2012;143(1-3):208-13. doi: <http://dx.doi.org/10.1016/j.jad.2012.05.056>. PubMed PMID: Peer Reviewed Journal: 2012-20550-001.

109. Pineda DA, Guerrero OL, Pinilla ML, Estupinan M. Usefulness of a screening questionnaire for post traumatic stress in a Colombian population [Spanish] Utilidad de un cuestionario para rastreo del estrés postraumático en una población colombiana. Revista de Neurologia. 2002;34(10):911-6.

110. Pinho MX, Custodio O, Makdisse M, Carvalho ACC. Reliability and validity of the geriatric depression scale in elderly individuals with coronary artery disease. [Portuguese]

Confiabilidade e validade da escala de depressao geriatrica em idosos com doenca arterial coronariana. Arquivos Brasileiros de Cardiologia. 2010;94(5):570-9. PubMed PMID: 2010347663.

111. Pitanupong J, Liabsuetrakul T, Vittayanont A. Validation of the Thai Edinburgh Postnatal Depression Scale for screening postpartum depression. Psychiatry Research. 2007;149(1-3):253-9. doi: <http://dx.doi.org/10.1016/j.psychres.2005.12.011>. PubMed PMID: Peer Reviewed Journal: 2007-02898-028.

112. Pollock JI, Manaseki-Holland S, Patel V. Detection of depression in women of child-bearing age in non-western cultures: A comparison of the Edinburgh Postnatal Depression Scale and the Self-Reporting Questionnaire-20 in Mongolia. Journal of Affective Disorders 2006;92(2-3):267-71. doi: <http://dx.doi.org/10.1016/j.jad.2006.02.020>. PubMed PMID: Peer Reviewed Journal: 2006-06957-015.

113. Puertas G, Patel V, Marshall T. Are visual measures of mood superior to questionnaire measures in non-Western settings? Soc Psychiatry Psychiatr Epidemiol. 2004;39:662-6.

114. Pupo MC, Jorge MR, Schoedl AF, Bressan RA, Andreoli SB, Mello MF, et al. The accuracy of the Clinician-Administered PTSD Scale (CAPS) to identify PTSD cases in victims of urban violence. Psychiatry Research. 2011;185(1-2):157-60. doi: <http://dx.doi.org/10.1016/j.psychres.2009.11.006>.

115. Regmi S, Sligl W, Carter D, Grut W, Seear M. A controlled study of postpartum depression among Nepalese women: validation of the Edinburgh Postpartum Depression Scale in Kathmandu. Tropical Medicine and International Health. 2002;7(4):378-82. doi: <http://dx.doi.org/10.1046/j.1365-3156.2002.00866.x>.

116. Rochat TJ, Tomlinson M, Newell ML, Stein A. Detection of antenatal depression in rural HIV-affected populations with short and ultrashort versions of the Edinburgh Postnatal Depression Scale (EPDS). Archives of Women's Mental Health. 2013;16(5):401-10. doi: <http://dx.doi.org/10.1007/s00737-013-0353-z>.

117. Rosliwati MY, Rohayah H, Jamil BYM, Zaharah S. Validation of the Malay Version of Children Depression Inventory (CDI) among Children and Adolescents attending Outpatient Clinics in Kota Bharu, Kelantan. Malaysian Journal of Psychiatry. 2008;17(1).

118. Rueda-Jaimes GE, Camacho Lopez PA, Rangel-Martinez-Villalba AM. Validation of two short versions of the Centre for Epidemiological Studies Depression Scale in Colombian adolescents. [Spanish]

Validacion de dos versiones cortas de la escala para depresion del Centro de Estudios Epidemiologicos en adolescentes colombianos. Atencion Primaria. 2009;41(5):255-61. doi: <http://dx.doi.org/10.1016/j.aprim.2008.09.005>. PubMed PMID: 2009244041.

119. Rumble S, Swartz L, Parry C, Zwarenstein M. Prevalence of psychiatric morbidity in the adult population of a rural South African village. Psychological Medicine. 1996;26(5):997-1007. PubMed PMID: 1996283804.

120. Russell PS, Basker M, Russell S, Moses PD, M.K CN, Minju KA. Comparison of a self-rated and a clinician-rated measure for identifying depression among adolescents in a primary-care setting. Indian Journal of Pediatrics. 2012;79(SUPPL. 1):S45-S51. doi: <http://dx.doi.org/10.1007/s12098-011-0438-5>. PubMed PMID: 2012092942.

121. Saipanish R, Lotrakul M, Sumrithe S. Reliability and validity of the Thai version of the WHO-Five Well-Being Index in primary care patients. Psychiatry and Clinical Neurosciences. 2009;63(2):141-6. doi: <http://dx.doi.org/10.1111/j.1440-1819.2009.01933.x>. PubMed PMID: Peer Reviewed Journal: 2009-04302-002.

122. Salinas-Rodriguez A, Manrique-Espinoza B, Acosta-Castillo I, Tellez-Rojo MM, Franco-Nunez A, Gutierrez-Robledo LM, et al. Validation of a cutoff for the Depression Scale of the Center for Epidemiologic Studies, Brief Version (CESD-7). Salud Publica de Mexico. 2013;55(3):267-74.

123. Salle E, Rocha NS, Rocha TS, Nunes C, Chaves MLF. Depression rating scales as screening tools for depression in high school students

Escalas psicometricas como instrumentos de rastreamento para depressao em estudantes do ensino medio. Revista de Psiquiatria Clinica. 2012;39(1):24-7. doi: <http://dx.doi.org/10.1590/S0101-60832012000100005>. PubMed PMID: 2012227922.

124. Santos IS, Matijasevich A, Tavares BF, da Cruz Lima AC, Riegel RE, Lopes BC. Comparing validity of Edinburgh scale and SRQ20 in screening for post-partum depression. Clinical Practice and Epidemiology in Mental Health. 2007;3(18). doi: <http://dx.doi.org/10.1186/1745-0179-3-18>. PubMed PMID: 2008230916.

125. Santos IS, Tavares BF, Munhoz TN, de Almeida LSP, da Silva NTB, Tams BD, et al. Sensitivity and specificity of the Patient Health Questionnaire-9 (PHQ-9) among adults from the general population. [Portuguese] Sensibilidade e especificidade do Patient Health Questionnaire-9 (PHQ-9) entre adultos da população geral. Cadernos de Saude Publica. 2013;29(8):1533-43.

126. Scazufca M, Menezes PR, Vallada H, Araya R. Validity of the self reporting questionnaire-20 in epidemiological studies with older adults: Results from the Sao Paulo Ageing & Health Study. Social Psychiatry and Psychiatric Epidemiology 2009;44(3):247-54. doi: <http://dx.doi.org/10.1007/s00127-008-0425-y>. PubMed PMID: Peer Reviewed Journal: 2009-02645-010.

127. Segopolo MT, Selemogwe MM, Plattner IE, Ketlogetswe N, Feinstein A. A screening instrument for psychological distress in Botswana: Validation of the Setswana version of the 28-item general health questionnaire. International Journal of Social Psychiatry. 2009;55(2):149-56. doi: <http://dx.doi.org/10.1177/0020764008093448>. PubMed PMID: Peer Reviewed Journal: 2009-03745-005.

128. Shamasunder C, Sriram TG, Murali Raj SG, Shanmugham V. Validity of a short 5-item version of the General Health Questionnaire (G.H.Q.). Indian Journal of Psychiatry. 1986;28(3):217-9. PubMed PMID: Peer-Reviewed Status-Unknown: 1991-55824-001.

129. Sheng L. Better detection of non-psychotic mental disorders by case description method in China. Asian Journal of Psychiatry. 2010;3(4):227-32. doi: <http://dx.doi.org/10.1016/j.ajp.2010.07.011>. PubMed PMID: 2010672115.

130. Sherina M, Arroll B, Goodyear-Smith F, Zain AMD. Screening for depression with a brief questionnaire in a primary care setting: Validation of the two questions with help question (Malay version). International Journal of Psychiatry in Medicine. 2011;41(2):143-54. doi: <http://dx.doi.org/10.2190/PM.41.2.d>. PubMed PMID: 2011224505.

131. Sherina M, Arroll B, Goodyear-Smith F. Criterion validity of the PHQ-9 (Malay version) in a primary care clinic in Malaysia. Medical Journal of Malaysia. 2012;67(3):309-15. PubMed PMID: 2012453498.

132. Sherina MS, Arroll B, Goodyear-Smith F. Validation of the GAD-7 (Malay version) among women attending a primary care clinic in Malaysia. J Prim Health Care. 2012;4(1):5-11.

133. Sibai AM, Chaaya M, Tohme RA, Mahfoud Z, Al-Amin H. Validation of the Arabic version of the 5-item WHO Well Being Index in elderly population. International Journal of Geriatric Psychiatry. 2009;24(1):106-7. doi: <http://dx.doi.org/10.1002/gps.2079>. PubMed PMID: Peer Reviewed Journal: 2009-01067-014.

134. Spies G, Stein DJ, Roos A, Faure SC, Mostert J, Seedat S, et al. Validity of the Kessler 10 (K-10) in detecting DSM-IV defined mood and anxiety disorders among pregnant women. Archives of Women's Mental Health. 2009;12(2):69-74. doi: <http://dx.doi.org/10.1007/s00737-009-0050-0>. PubMed PMID: Peer Reviewed Journal: 2009-04892-002.

135. Spies G, Kader K, Kidd M, Smit J, Myer L, Stein DJ, et al. Validity of the K-10 in detecting DSM-IV-defined depression and anxiety disorders among HIV-infected individuals. AIDS Care. 2009;21(9):1163-8.

136. Stewart RC, Kauye F, Umar E, Vokhiwa M, Bunn J, Fitzgerald M, et al. Validation of a Chichewa version of the self-reporting questionnaire (SRQ) as a brief screening measure for maternal depressive disorder in Malawi, Africa. Journal of Affective Disorders. 2009;112(1-3):126-34. doi: <http://dx.doi.org/10.1016/j.jad.2008.04.001>. PubMed PMID: 18504058.

137. Suttajit S, Srisurapanont M, Pilakanta S, Charnsil C, Suttajit S. Reliability and validity of the Thai version of the Calgary Depression Scale for Schizophrenia. Neuropsychiatric Disease and Treatment. 2013;9:113-8. PubMed PMID: Peer Reviewed Journal: 2013-07043-001.

138. Tan SMK, Loh SF, Adam Bujnag M, Haniff J, Abd Rahman FN, Ismail F, et al. Validation of the Malay version of children's depression inventory: International Medical Journal. 20 (2) (pp 188-191), 2013. Date of Publication: April 2013.; 2013.

139. Tesfaye M, Hanlon C, Wondimagegn D, Alem A. Detecting postnatal common mental disorders in Addis Ababa, Ethiopia: validation of the Edinburgh Postnatal Depression Scale and Kessler Scales. Journal of Affective Disorders. 2010;122(1-2):102-8. doi: <http://dx.doi.org/10.1016/j.jad.2009.06.020>.

140. Tran TD, Tran T, Fisher J. Validation of three psychometric instruments for screening for perinatal common mental disorders in men in the north of Vietnam. Journal of Affective Disorders. 2012;136(1/2):104-9. doi: <http://dx.doi.org/10.1016/j.jad.2011.08.012>.

141. Tran TD, Tran T, Fisher J. Validation of the depression anxiety stress scales (DASS) 21 as a screening instrument for depression and anxiety in a rural community-based cohort of northern Vietnamese women. BMC Psychiatry. 2013;13. doi: <http://dx.doi.org/10.1186/1471-244X-13-24>.

142. Tuan T, Harpham T, Huong NT. Validity and reliability of the Self-reporting Questionnaire 20 items in Vietnam. Hong Kong Journal of Psychiatry. 2004;14(3):15-8+32-3. PubMed PMID: 2005295487.

143. Uwakwe R. Affective (depressive) morbidity in puerperal Nigerian women: validation of the Edinburgh postnatal depression scale. Acta Psychiatrica Scandinavica. 2003;107(4):251-9. doi: <http://dx.doi.org/10.1034/j.1600-0447.2003.02477.x>.

144. Vega-Dienstmaier JM, Mazzotti Suarez G, Campos Sanchez M. Validation of a Spanish version of the Edinburgh Postnatal Depression Scale. [Spanish] Validacion de una version en espanol de la Escala de Depresion Postnatal de Edimburgo. Actas espanolas de psiquiatria. 2002;30(2):106-11. PubMed PMID: 12028943.

145. Vittayanont A, Liabsuetrakul T, Pitanupong J. Development of Postpartum Depression Screening Scale (PDSS): a Thai version for screening postpartum depression. Journal of the Medical Association of Thailand. 2006;89(1):1-7.

146. Wang Y, Guo X, Lau Y, Chan KS, Yin L, Chen J. Psychometric evaluation of the Mainland Chinese version of the Edinburgh Postnatal Depression Scale. International Journal of Nursing Studies. 2009;46(6):813-23. doi: <http://dx.doi.org/10.1016/j.ijnurstu.2009.01.010>. PubMed PMID: Peer Reviewed Journal: 2009-06767-009.

147. Weobong B, Akpalu B, Doku V, Owusu-Agyei S, Lisa Hurt L, Kirkwood B, et al. The comparative validity of screening scales for postnatal common mental disorder in Kintampo, Ghana. Journal of Affective Disorders. 2009;113:109-17.

148. Wulsin L, Somoza E, Heck J. The feasibility of using the Spanish PHQ-9 to screen for depression in primary care in Honduras. Primary Care Companion J Clin Psychiatry. 2002;4(5):191-5.

149. Xiao W, Liu H, Zhang H, Liu Q, Fu P, Chen J, et al. Reliability and validity of the Chinese version of the Calgary Depression Scale for Schizophrenia. Australian and New Zealand Journal of Psychiatry. 2009;43(6):548-53. doi: <http://dx.doi.org/10.1080/00048670902873672>. PubMed PMID: 2009398231.

150. Yang Y, Ding R, Hu D, Zhang F, Sheng L. Reliability and validity of a chinese version of the hads for screening depression and anxiety in psycho-cardiological outpatients. Comprehensive Psychiatry. 2014;55:215-20. doi: <http://dx.doi.org/10.1016/j.comppsych.2013.08.012>. PubMed PMID: Peer Reviewed Journal: 2013-38971-001.

151. Zhang YL, Liang W, Chen ZM, Zhang HM, Zhang JH, Weng XQ, et al. Validity and reliability of Patient Health Questionnaire-9 and Patient Health Questionnaire-2 to screen for depression among college students in China. Asia-Pacific Psychiatry. 2013;5(4):268-75. doi: <http://dx.doi.org/10.1111/appy.12103>.

152. Zhou H, Zhao Z, Qiu CH. Application of the two-item patient health questionnaire depression scale in screening for post-stroke depression. [Chinese]. Chinese Journal of Cerebrovascular Diseases 2013;10(5):255-8. doi: <http://dx.doi.org/10.3969/j.issn.1672-5921.2013.05.007>.

153. Zubaran C, Foresti K, Schumacher MV, Amoretti AL, Muller LC, Thorell MR, et al. Validation of a screening instrument for postpartum depression in Southern Brazil. Journal of Psychosomatic Obstetrics & Gynecology. 2009;30(4):244-54. doi: <http://dx.doi.org/10.3109/01674820903254724>. PubMed PMID: Peer Reviewed Journal: 2010-11973-006.
