# Supplementary material for: Validated Screening Tools for Common Mental Disorders in Low and Middle Income Countries: A Systematic Review
Source: PLoS One. 2016 Jun 16;11(6):e0156939. doi: 10.1371/journal.pone.0156939 (PMC4911088; doi:10.1371/journal.pone.0156939)
Supplement: S4 File — Acronyms for all screening tools listed in the results database. (DOCX) [file pone.0156939.s004.docx]

**S4 File. Screening Tool Acronyms**

The following is a complete list of screening tool acronyms included in S1 File. Results Database.

AVIDI Akena's visual depression inventory

AYMH Arab youth mental health scale

BAI Beck anxiety inventory

BDI Beck depression inventory

BDI-PC Beck depression inventory for primary care

BDI-SF Beck depression inventory short form

BSPS Brief social phobia scale

CAPS Clinician-administered PTSD scale

CDI Children’s depression inventory

CDRS Children′s depression rating scale

CDSS Calgary depression scale for schizophrenia (CDSS-C = Chinese version)

CES-D Center for Epidemiological Studies depression scale

CES-DC Center for Epidemiological Studies depression scale for children

CGHQ Chile scoring method for the general health questionnaire

CPDS Child psychosocial distress screener

C-PDSS Chinese version of the postpartum depression screening scale

CPSS Child PTSD symptom scale

CRS Carroll rating scale

DASS Depression, anxiety and stress scale

DSRS Depression self-rating scale

EPDS Edinburgh postnatal depression scale

Euro-D European depression scale

GAD Generalised anxiety disorder scale

GDS Geriatric depression scale

GHQ General health questionnaire

HADS Hospital anxiety and depression scale

HAM-D Hamilton rating scale for depression

HSCL Hopkins symptom checklist

HTQ Harvard trauma questionnaire

IES Impact of events scale

K Kessler psychological distress scale

MDI Major depression inventory

NDDI-E Neurological disorders depression inventory for epilepsy

PADQ Pakistan anxiety and depression questionnaire

PDS Peradeniya Depression Scale

PDS Posttraumatic diagnostic scale

PDSS Postpartum Depression Screening Scale

PHQ Patient health questionnaire

SPIN Social phobia inventory

SRQ Self-report questionnaire

TQWHQ Two questions with help question

TSSC Traumatic stress symptom checklist

WHO-5 WHO wellbeing index (WHO-5-A = Arabic version; WHO-5-T = Thai version)

ZSAS Zung's self-rated anxiety scale

ZSDS Zung's self-rated depression scale

Notes

- The addition of a number after the acronym indicates that multiple versions of the tool are available with different numbers of questions. For example, the PHQ-2 asks just the first two of the PHQ-9’s nine questions.
- The addition of -R or -r after the acronym indicates a revised/updated version of the original tool.
- The addition of -A or -D after the acronym indicates that only the anxiety or depression subscale was used.
- The addition of Mini- before the acronym indicates an abbreviated version of the tool.
